# Identifying Putative Resistance Genes for Barley Yellow Dwarf Virus-PAV in Wheat and Barley

**DOI:** 10.3390/v15030716

**Published:** 2023-03-09

**Authors:** Glenda Alquicer, Emad Ibrahim, Midatharahally N. Maruthi, Jiban Kumar Kundu

**Affiliations:** 1 Plant Virus and Vector Interactions-Centre for Plant Virus Research, Crop Research Institute, Drnovská 507, 161 06 Prague, Czech Republic; 2 Agriculture, Health and Environment Department, Natural Resources Institute, University of Greenwich, Medway Campus, Chatham, Kent ME4 4TB, UK; 3 Laboratory of Virology-Centre for Plant Virus Research, Institute of Experimental Botany of the Czech Academy of Sciences, Rozvojová 263, 165 02 Prague, Czech Republic

**Keywords:** BYDV-PAV, wheat, barley, gene expression, RT-qPCR, resistance

## Abstract

Barley yellow dwarf viruses (BYDVs) are one of the most widespread and economically important plant viruses affecting many cereal crops. Growing resistant varieties remains the most promising approach to reduce the impact of BYDVs. A Recent RNA sequencing analysis has revealed potential genes that respond to BYDV infection in resistant barley genotypes. Together with a comprehensive review of the current knowledge on disease resistance in plants, we selected nine putative barley and wheat genes to investigate their involvement in resistance to BYDV-PAV infection. The target classes of genes were (i) nucleotide binding site (NBS) leucine-rich repeat (LRR), (ii) coiled-coil nucleotide-binding leucine-rich repeat (CC-NB-LRR), (iii) LRR receptor-like kinase (RLK), (iv) casein kinase, (v) protein kinase, (vi) protein phosphatase subunits and the transcription factors (TF) (vii) MYB TF, (viii) GRAS (gibberellic acid-insensitive (GAI), repressor of GAI (RGA) and scarecrow (SCR)), and (ix) the MADS-box TF family. Expression of genes was analysed for six genotypes with different levels of resistance. As in previous reports, the highest BYDV-PAV titre was found in the susceptible genotypes Graciosa in barley and Semper and SGS 27-02 in wheat, which contrast with the resistant genotypes PRS-3628 and Wysor of wheat and barley, respectively. Statistically significant changes in wheat show up-regulation of *NBS-LRR*, *CC-NBS-LRR* and *RLK* in the susceptible genotypes and down-regulation in the resistant genotypes in response to BYDV-PAV. Similar up-regulation of *NBS-LRR*, *CC-NBS-LRR*, *RLK* and *MYB TF* in response to BYDV-PAV was also observed in the susceptible barley genotypes. However, no significant changes in the expression of these genes were generally observed in the resistant barley genotypes, except for the down-regulation of *RLK*. *Casein kinase* and *Protein phosphatase* were up-regulated early, 10 days after inoculation (dai) in the susceptible wheat genotypes, while the latter was down-regulated at 30 dai in resistant genotypes. *Protein kinase* was down-regulated both earlier (10 dai) and later (30 dai) in the susceptible wheat genotypes, but only in the later dai in the resistant genotypes. In contrast, *GRAS TF* and *MYB TF* were up-regulated in the susceptible wheat genotypes while no significant differences in *MADS TF* expression was observed. *Protein kinase*, *Casein kinase* (30 dai), *MYB TF* and *GRAS TF* (10 dai) were all up-regulated in the susceptible barley genotypes. However, no significant differences were found between the resistant and susceptible barley genotypes for the *Protein phosphatase* and *MADS FT* genes. Overall, our results showed a clear differentiation of gene expression patterns in both resistant and susceptible genotypes of wheat and barley. Therefore, further research on *RLK, NBS-LRR, CC-NBS-LRR, GRAS TF* and *MYB TF* can lead to BYDV-PAV resistance in cereals.

## 1. Introduction

The barley yellow dwarf viruses (BYDVs) complex causes one of the most economically important viral diseases in cereals worldwide and leads to significant yield losses in cereal crops such as wheat, barley, rice, maize and oats [1,2]. BYDV-PAV (Genus *Luteovirus*), the most prevalent and damaging species of BYDVs, is transmitted by at least 25 species of aphid vectors and almost all plants of the *Poaceae* family can be infected, making over 150 species potential sources of infection [2,3]. Several strategies have been proposed to mitigate the devastating impact of BYDV-PAV on all major cereal crops worldwide. Control methods for the phloem-limited luteovirus include spraying insecticides to reduce aphid populations. However, a more economically and environmentally effective approach to control is to grow tolerant or resistant varieties [4]. Resistance is defined as a compatible host–virus interaction in which the virus may or may not replicate to some extent in the host, but invasion is limited compared to a susceptible host and symptoms are localised or absent altogether [5]. Host resistance is either tolerance—when symptoms and yield losses are reduced but virus replication remains unchanged—or resistance—when both virus replication and symptoms are reduced [6]. Resistance to BYDV-PAV has been studied in wheat (*Triticum aestivum* L.) and barley (*Hordeum vulgare* L.) [7,8,9]. Several genes for BYDV-PAV tolerance were identified in wheat (*Bdv*1, *Bdv*2, *Bdv*3 and *Bdv*4) [10,11], and barley (*Ryd*1 [12], *Ryd*2 [13], *Ryd*3 [14] and *Ryd*4 [15]). However, breeding for resistant varieties has shown that different levels of resistance can be achieved and that not all genes have been effectively introduced so far (reviewed in [16]). The most effective genes that have been successfully introduced are *Ryd*2 in barley varieties (e.g., Atlas68, Wysor, Wbon, Travira) and *Bdv*2 in wheat varieties (e.g., Mackellar, Glover), which have shown high levels of resistance to BYDV-PAV [7,8,16]. These resistance phenotypes correspond to a low visual symptom score (VSS), a reduction in green grain weight per spike (GRS -R) and a low virus titre [4,7,8].

Transcriptome analyses of plants in response to viral infections reveal a complex relationship between genes and their regulation [17]. The transcriptome profile and expression of target genes are unique and restricted to specific host-virus interactions [18], depending on pathogenicity determinants of the virus that recognise and interact with host-specific proteins encoded by *R* genes [19,20] or via signal transduction pathways [21]. Both trigger a plant defence response leading to host susceptibility or resistance. Recently, a meta-analysis of resistance genes (*R*) distinguished nine direct and indirect molecular mechanisms by which *R* proteins can promote or trigger disease resistance [22]. These include (i) recognition of pathogenic molecules on the cell surface by receptor-like proteins and receptor-like kinases; (ii) intracellular recognition of pathogenic molecules by nucleotide binding site (*NBS)* or Leucine-rich receptors (*LRR)* or by integrated domains; and (iii) perception of transcriptional activator-like effectors. To cover this range, we selected nine genes from the predicted expression patterns of miRNA from barley genotypes associated with resistance to BYDV-PAV [23] to assess their expression in both barley and wheat genotypes. RT-qPCR-based gene expression analysis [24] was also performed on both infected and uninfected genotypes with different levels of resistance. Another factor we focused on was the gene expression profile at early and late stages of virus infection. We compared transcriptome results with the viral titre of each genotype to better correlate gene expression in the context of plant defence and resistance against BYDV-PAV. The gene expression analysis has revealed at least four (*NBS*, *CC-NBS*, *RLK*, *MYB TF* and *GRAS TF*) of the investigated genes associated with wheat and barley resistance to BYDV-PAV. These genes could be potential targets in improving BYDV-PAV resistance in cereals. The gene expression profiles and methods described could also be used as an effective tool for assessing the resistance of cereals to BYDV-PAV.

## 2. Materials and Methods

### 2.1. Plant Growth and Virus Inoculation

Six barley (*H. vulgare*) genotypes were selected: Graciosa, as a susceptible control; Wysor, Wbon and Travira, carrying *Ryd*2, as resistant genotypes; and the breeding lines Vir8:3 and Vir13:8 as crosses between six-row non-malting winter barley genotypes. Similarly, six wheat genotypes (*T. aestivum*) were included: SGS 27-02 and Semper as susceptible controls; Tobak as a winter-tolerant but drought-sensitive genotype; and Sparta, Elan and PSR 3628 with conferred resistance. Barley and wheat seeds were planted in 10 × 10 cm plastic pots filled with a premixed sterilised substrate, one plant per pot. Aphids (*Rhopalospiphum padi*) were used for virus transmission [23]. Before the experiment, half the aphids were kept on virus-free plants and the other half were kept on BYDV-PAV- infected (GenBank accession number FJ645745) [25] barley plants for one week to acquire the virus. Fourteen-day-old plants were then inoculated with BYDV-PAV using the viruliferous aphids for 3 days. Plants were treated with a dose of the insecticide acetamiprid (0.25 mL/L H_2_O) to control the aphids’ vector. Plants were incubated at 21 °C, 16 h of light and 60% humidity in a greenhouse in separate insect-proof net cages for symptom expression. Three leaf samples were collected for each genotype, each treatment (control and infection) and at two time points: 10 and 30 days after inoculation (dai). A total of 72 samples were collected for each genotype, ground in liquid nitrogen and stored in aliquots of 100 mg at −80 °C.

### 2.2. RNA Isolation and cDNA Preparation

Total RNA was isolated using Trizol-based reagent RNA blue kit (Top-Bio, Vestec, Czech Republic) and purified using RNA Clean and Concentrator-5 (Zymo Research, Ir-vine, CA, USA) according to the manufacturer’s protocol. The concentration and purity of the isolated RNA was measured spectrophotometrically (NanoDrop 2000; Thermo Scientific, Wilmington, DE, USA). Complementary DNA was synthesised using 1 µg of total RNA, RevertAid reverse transcriptase 200 U/µL and oligo(dT)_18_ (for gene expression analysis) or random hexamer primer (for virus titre analysis) (Thermo Scientific, Waltham, MA, USA) according to the manufacturer’s instructions.

### 2.3. Analysis of BYDV-PAV Titre in Wheat and Barley Plants by RT-qPCR

Quantification of the BYDV-PAV titre required a 5-fold dilution of the cDNA for the qPCR assays in a LightCycler 480 (Roche, Basel, Switzerland). The BYDV-PAV-positive control sample was prepared according to Jarošová and Kundu [24] and a tenfold diluted clone was used to obtain the standard curve (8 points). The number of viral copies in the standard sample was calculated using the formula: number of copies = (amount of DNA × 6.022 × 10^23^)/(length of the plasmid × 1 × 10^9^ × 660) [26,27]. We obtained the Ct values by absolute quantification/2nd derivative maximum (Light Cycler 480 software; Roche, Basel, Switzerland) against the logarithm of the calculated copy numbers for each standard dilution. A linear regression analysis (Microsoft Excel) was performed on the Ct values to determine BYDV-PAV titre in all barley and wheat samples by fitting the Ct values to the standard curve. Standards and samples were measured in triplicate. Cycling conditions were as in [28]: 10 min at 95 °C for initial denaturation; 40 cycles of amplification (5 s at 95 °C, 30 s at 60 °C and 20 s at 72 °C). The PCR reaction consisted of primers PVinterF [24] and YanRA [29] (0.42 μM) (Supplementary Table S1), 6 μL of 2x LightCycler 480 SYBR Green I Master (Roche, Basel, Switzerland), 1 μL cDNA or standard samples and sterile nuclease-free water to reach a final volume of 12 μL. The estimated BYDV-PAV copy number values per μL plant DNA per genotype at 10 and 30 days were analysed using GraphPad Prism 9 software (San Diego, CA, USA).

### 2.4. Selection of Genes and Analysis of Gene Expression by RT-qPCR

The target genes for this study were selected based on analysis of expression patterns of miRNA in response to BYDV-PAV [23]. Jarošová et al. [23] identified known and novel miRNA associated with defence-related genes or transcription factors regulating the stress response. Based on those findings and according to the current understanding of the disease resistance in plants [9,22,30], we selected nine putative barley and wheat genes that include: (i) *NBS-LRR*, (ii) *CC-NB-LRR* class, (iii) *RLK*, (iv) *casein kinase*, (v) *protein kinase* genes, (vi) *protein phosphatase* subunits and the transcription factors (TF) (vii) *MYB* superfamily, (viii) *GRAS* and (ix) *MADS -box TF*. The sequences of the selected genes are available in the NCBI database (GenBank accession numbers in Supplementary Table S1) for both barley and wheat. Primers for our target genes were designed using primer-BLAST (NCBI; RRID:SCR_003095) with melting temperatures between 59 °C and 61 °C and amplicon lengths between 70 and 190 bp. The other parameters were left at the default setting. We allowed a maximum of two mismatches between the primer and the target sequence and then carefully checked each base in the primer sequence to avoid mismatches, especially in the last five nucleotides of the 3’ end. The final oligos were purchased from Eurofins Genomics (Eurofins, Val Fleuri, Luxemburg). Two primer sets were designed for each gene and the pairs with an efficiency closer to 2 were selected for further analysis. PCR efficiency values (E) were calculated for each gene from the given slope after standard curves (10-fold dilutions of pooled cDNA samples) were generated using the formula E (%) = (−1/(10^slope^ − 1)) × 100 and considering 100% = 2 [31]. Two reference genes proposed by Jarošová et al. [24], *TubB* and *GAPDH*, were used in this study. Amplifications were also performed with a LightCycler 480 instrument II (Roche, Basil, Switzerland) in 384-well plates with 12 μL reaction solutions per well using 6 μL LightCycler^®^ 480 SYBR Green I Master 2x concentrated mixture forward and reverse primers (0.42 μM), 5 μL cDNA template (diluted 10-fold). Cycling conditions were: 95 °C for 10 min, followed by 45 cycles of 95 °C for 5 s and 60 °C for 30 s and 72 °C for 10 s. To check reproducibility, each assay was performed with three technical replicates for each of the three biological samples. The resulting Ct values were normalised to the expression of the reference genes to calculate the double delta Ct value (2^−ΔΔCT^) to obtain the change in gene expression [32]. The expression change for each gene per genotype at 10 and 30 dai was further analysed using GraphPad Prism 9 software (San Diego, CA, USA).

## 3. Results

### 3.1. The BYDV-PAV Titre in Wheat and Barley Genotypes

**Figure 1:**
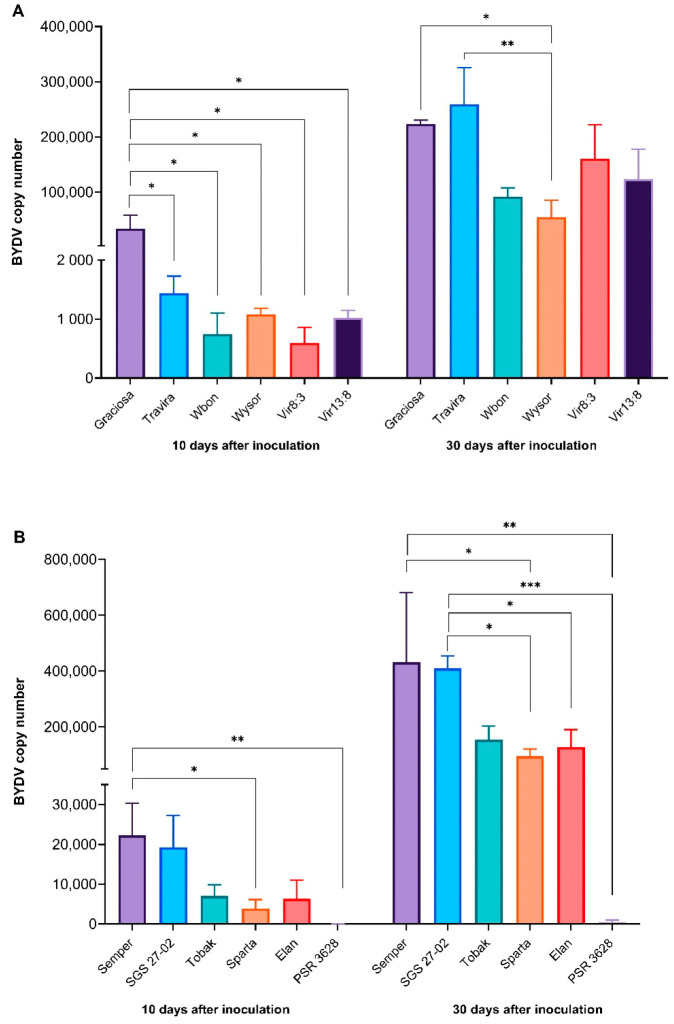
BYDV-PAV titre (virus copy number) in barley genotypes (**A**) and wheat genotypes (**B**). Significant differences are shown after one-way ANOVA and Tukey’s multiple comparison test, (* = *p* < 0.05 ** = *p* < 0.01 and *** = *p* < 0.001). Bars represent the means and standard errors of three biological replicates.

BYDV-PAV was detected in all barley and wheat plants at 10 and 30 dai by RT-qPCR. One-way ANOVA revealed a statistically significant difference [F (11, 16) = 2.695], *p* = 0.0352 in barley genotypes at 10 dai (Figure 1A). Tukey’s test for multiple comparisons also revealed that the high BYDV-PAV titre seen in the susceptible genotype Graciosa was significantly different to all barley genotypes (*p* < 0.05, 95% C.I. = 738 to 65,656). At 30 dai, all genotypes had elevated BYDV-PAV titres, but significant differences were still observed in one-way ANOVA [F (11, 23) = 8.2772], *p* < 0.0001. Graciosa and Travira had the highest titres, but Tukey’s test for multiple comparisons revealed significant differences only between Graciosa and Wysor genotypes (*p* < 0.05, 95% C.I. = 757 to 336,929); and Travira and Wysor (*p* < 0.01, 95% C.I. = 36,747 to 372,919) (Supplementary Figure S1).

Between the wheat genotypes, the susceptible Semper and SGS 27-02 had the highest titres while the resistant PSR 3628 consistently registered the lowest BYDV-PAV titre. The one-way ANOVA showed significant differences at both 10 dai [F (11, 21) = 4681], *p* < 0,001, and 30 dai [F (11, 21) = 7566], *p* < 0.0001 (Figure 1B). Tukey’s test for multiple comparisons found significantly higher BYDV-PAV titre in Semper compared to Sparta (*p* < 0.05, 95% C.I. = 763 to 35,974 at 10 dai, and *p* < 0.05, 95% C.I. = 29,115 to 644,644 at 30 dai) and PSR 3628 (*p* < 0.01, 95% C.I. = 4594 to 39,804 at 10 dai, and *p* < 0.01, 95% C.I. = 123,266 to 738,796 at 30 dai). The same test, at 30 dai, also found significantly higher titre in SGS 27-02 compared to Elan (*p* < 0.05, 95% C.I. = 7065 to 557,611), Sparta (*p* < 0.05, 95% C.I. = 39,638 to 590,184), and PSR 3628 (*p* < 0.001, 95% C.I. = 133,789 to 684,335).

### 3.2. Correlation between Gene Expression and Resistance to BYDV-PAV in Wheat and Barley

**Figure 2:**
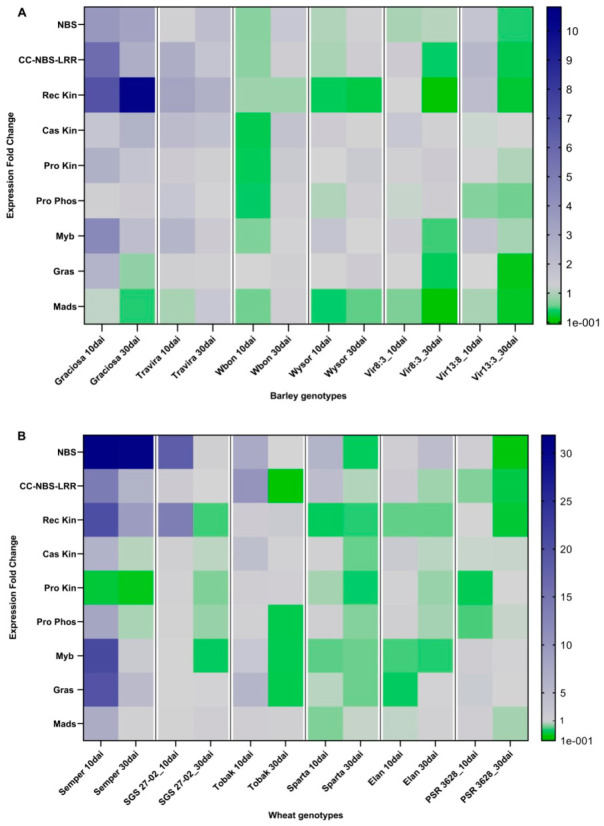
Heat map showing expression profile of genes associated with resistance to BYDV-PAV infection in barley genotypes (**A**) and wheat genotypes (**B**). The blue colour stands for upregulation, the green for downregulation and the grey for control values (1). Up-regulation is mainly observed in susceptible genotypes (**left**), while down-regulation is more common in resistant genotypes (**right**). These patterns are particularly consistent for NBS, CC-NBS-LRR and Rec Kin in the inoculated samples of barley and wheat. Slight differences between 10 dai and 30 dai are also observed. The maps were generated with Graphpad Prism software using expression fold change values obtained from qPCR, followed by statistical analysis using two-way ANOVA.

To characterise the gene expression profile in response to BYDV-PAV infection between genotypes with different levels of resistance, a heat map was constructed for barley and wheat using expression fold change (EFC) data from RT-qPCR (Figure 2). The maps for barley and wheat show different down-regulation and up-regulation profiles between genotypes and between 10 and 30 dai. The multi-factorial ANOVA analysis revealed a statistically significant interaction between the BYDV-PAV infection of barley genotypes (10 and 30 dai) and the expression fold change of the genes of interest F (88, 189) = 7.15, *p* < 0.0001, as well as a statistically significant interaction between BYDV-PAV infection in the wheat genotypes (10 and 30 dai) and the expression fold change of the genes of interest F (88, 201) = 12.42, *p* < 00001. In addition, Supplementary Table S1 shows heat maps with mean EFC, SEM and TTest analyses comparing control and infected samples for each case. The up- and down-regulation observed in the Vir8:13 and Vir13:8 genotypes confirm the involvement of these genes predicated by Jarošová et al. [23].

**Figure 3:**
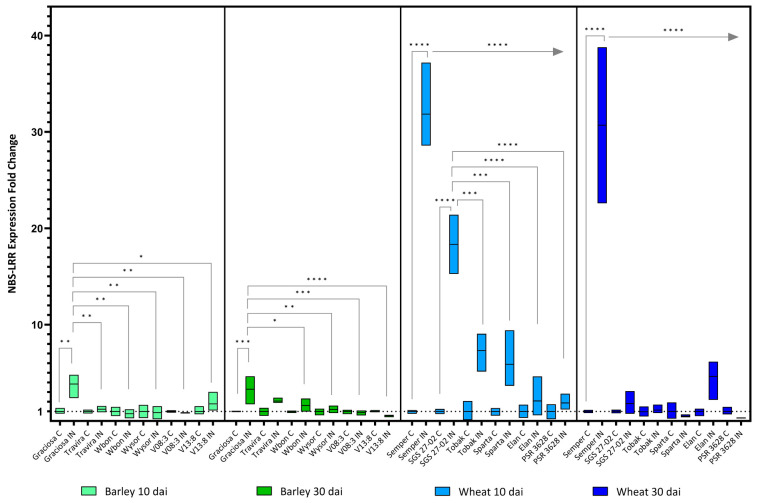
Box plots showing fold change in NBS-LRR resistance genes expression in different barley and wheat genotypes at 10 and 30 dai. The horizontal line in each box represents the mean expression fold change, and the lower and upper box limits the minimum and maximum values, respectively. Two-way ANOVA and Tukey’s multiple comparison test revealed significant differences * = *p* < 0.05, ** = *p* < 0.01, *** = *p* < 0.001, and **** = *p* < 0.0001. The straight arrow from Semper to PSR 3628 indicates the same*p*value when Semper is compared to all genotypes.

A more detailed analysis using two-way ANOVA and Tukey’s multiple comparisons test on gene expression showed significant differences for the *NBS-LRR* resistance genes (*NBS*), s at both 10 and 30 dai. For barley genotypes, statistically significant interactions were observed between the genotypes and BYDV-PAV infection at both 10 [F (5, 21) = 4.55, *p* = 0.006] and 30 dai [F (5, 24) = 5.29, *p* = 0.002]. The analysis also showed statistically significant effect of the genotypes on *NBS* expression at 10 dai (*p* = 0.006) and at 30 dai (*p* = 0.002). BYDV-PAV infection also had a significant effect on *NBS* expression at 10 (*p* = 0.03) and at 30 dai (*p* = 0.002). The susceptible genotype Graciosa mainly contributed to these differences. The high *NBS* expression was maintained at 10 dai in all genotypes while mainly in the resistant genotypes Wbon, Wysor, V08:3 and V13:8 at 30 dai (Figure 3).Similarly, the analysis revealed a statistically significant interaction between the effects of wheat genotype and BYDV-PAV infection at both 10 [F (5, 22) = 42.37, *p* < 0.0001] and 30 dai [F (5, 22) = 26.66, *p * < 0.0001] (Figure 3). As for genotypes, there is a statistically significant effect on NBS expression at 10 dai (*p* < 0.0001) and at 30 dai (*p* < 0.0001). Similar results were obtained for the effect of BYDV-PAV infection on NBS expression at 10 and 30 dai (*p* < 0.0001). At 10 dai, the susceptible lines Semper and SGS 27-02 showed a dramatic increase in *NBS* expression, but only Semper maintained this increase until day 30.

**Figure 4:**
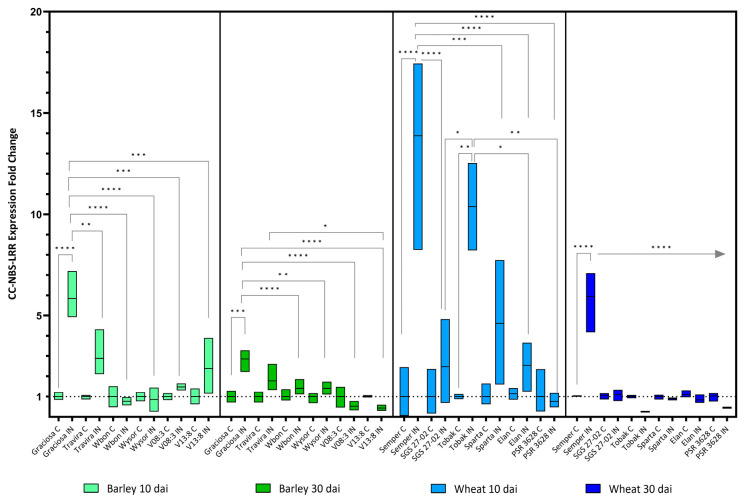
Box plots showing the expression fold change of CC-NBS-LRR in different barley wheat genotypes at 10 and 30 dai. The horizontal line in each box represents the mean expression fold change, and the lower and upper box limits the minimum and maximum values, respectively. Two-way ANOVA and Tukey’s multiple comparison test revealed significant differences * = *p* < 0.05, ** = *p* < 0.01, *** = *p* < 0.001, and **** = *p* < 0.0001.

The selected genes with conserved coil-coil nucleotide-binding site leucine-rich repeat motif (*CC-NBS-LRR*) also showed increased expression in the susceptible wheat and barley genotypes early after infection, but decreased by 30 days (Figure 4). The wheat genotype Tobak (winter tolerant but drought sensitive) showed a milder but still significant increase in *CC-NBS-LRR* expression only in the early phase (10 dai). [(Barley 10 dai BYDV-PAV -genotype interaction F (5, 21) = 8.95, *p* < 0.0001; genotype effect *p* < 0.0001; and BYDV-PAV effect *p* < 0.0001), (Barley 30 dai BYDV-PAV -genotype interaction F (5, 24) = 8.14, *p* < 0.0001; genotype effect *p* < 0.0001; and BYDV-PAV effect *p* = 0.004), (wheat 10 dai BYDV-PAV -genotype interaction F (5, 23) = 9.18, *p* < 0.0001; genotype effect *p* < 0.0001; and BYDV-PAV effect *p* < 0.0001) and (wheat 30 dai BYDV-PAV-genotype interaction F (5, 24) = 31.33, *p* < 0.0001; genotype effect *p* < 0.0001; and BYDV-PAV effect *p* = 0.002) according to the two-way test ANOVA with Tukey’s Multiple Comparison Test).

**Figure 5:**
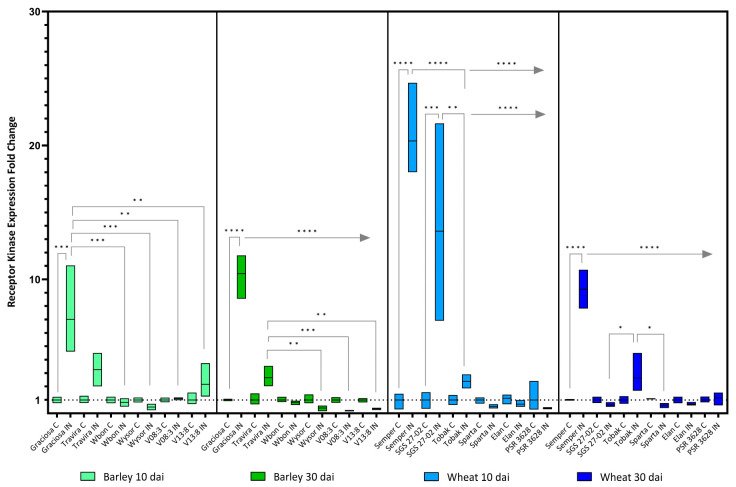
Box plots showing the expression fold change of LRR receptor-like kinase in different barley and wheat genotypes at 10 and 30 dai. The horizontal line in each box represents the mean expression fold change, and the lower and upper box limits the minimum and maximum values, respectively. Two-way ANOVA and Tukey’s multiple comparison test revealed significant differences * = *p* < 0.05, ** = *p* < 0.01, *** = *p* < 0.001, and **** = *p* < 0.0001. The straight line at the top indicates the same*p*value when that genotype is compared to the rest in line.

The expression of the LRR receptor-like kinase (Rec-Kin) genes examined in this study was significantly increased by 10 and 30 dai in the susceptible genotypes. In barley lines, two-way ANOVA showed statistically significant interactions between the effects of genotype and BYDV-PAV infection at 10 [F (5, 21) = 5.44, *p*= 0.002] and at 30 dai [F (5, 24) = 74.43, *p*< 0.0001]. The analysis also showed a statistically significant effect on the expression of Rec-kin at 10 (*p* = 0.002) and at 30 dai (*p* < 0.0001) depending on the genotype. BYDV-PAV infection had a significant effect on Rec Kin expression at 10 (*p* = 0.003) and 30 dai (*p* < 0.0001). In the susceptible genotype Graciosa, Rec Kin expression is significantly increased both at 10 dai compared to Wbon, Wysor, V08:3 and V13:8 and at 30 dai compared to all genotypes (Figure 5). Interestingly, the moderately resistant genotype Travira also showed a less pronounced but still significant increase in Rec Kin expression compared to Wysor, V08:3 and V13:8 at 30 dai. For wheat genotypes (Figure 5), the analysis revealed a statistically significant interaction between the effects of genotype and BYDV-PAV infection at 10 dai [F (5, 23) = 17.28, *p*< 0.0001] and 30 dai [F (5, 23) = 31.80, *p*< 0.0001]. Regarding genotypes, a statistically significant effect is seen on Rec Kin expression at 10 (*p* < 0.0001) and 30 dai (*p* < 0.0001). Similar results were found for the effect of BYDV-PAV infection on Rec-Kin expression at 10 and 30 dai (*p* < 0.0001). Shortly after inoculation (10 days), the susceptible lines Semper and SGS 27-02 showed a substantial significant increase in Rec-Kin expression, but their expression levels decreased at 30 dai and maintained a significantly high level only in Semper. The decrease was less pronounced in the Tobak genotype compared to some lines, but not compared to its control.

**Figure 6:**
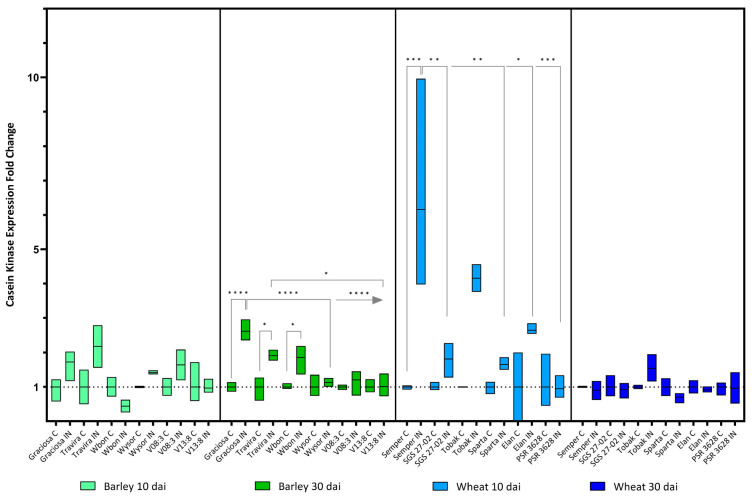
Box plots showing the expression fold change of Casein kinase in different barley and wheat genotypes at 10 and 30 dai. The horizontal line in each box represents the mean expression fold change, and the lower and upper box limits the minimum and maximum values, respectively. Two-way ANOVA and Tukey’s multiple comparison test revealed significant differences * = *p* < 0.05, ** = *p* < 0.01, *** = *p* < 0.001, and **** = *p* < 0.0001.

BYDV-PAV infection led to a significant increase in the expression of the *Casein kinase* gene in the genotypes Graciosa, Travira and Wbon only at 30 dai. However, the up-regulation was more significant in Graciosa and was almost three times higher compared to the infected genotypes Wysor, V08:3 and V13:3. In contrast, expression of the *Casein kinase*-like protein in the susceptible wheat genotype Semper increased as early at 10 dai but no significant changes were observed at 30 dai (Figure 6). (Barley 10 dai BYDV-PAV -genotype interaction F (5, 21) = 1.78, *p* = 0.161; genotype effect *p* = 0.161; and BYDV-PAV effect *p* = 0.004), (Barley 30 dai BYDV-PAV -genotype interaction F (5, 24) = 7.77, *p* = 0.0002; genotype effect *p* = 0.0002; and BYDV-PAV effect *p* < 0.0001), (wheat 10 dai BYDV-PAV -genotype interaction F (5, 23) = 4.82, *p* = 0.004; genotype effect *p* = 0.004; and BYDV-PAV effect *p* < 0.0001) and (wheat 30 dai BYDV-PAV -genotype interaction F (5, 24) = 1.88, *p* = 0.14; genotype effect not significant (ns); and BYDV-PAV effect p-value ns according to two-way ANOVA with Tukey’s multiple comparison test).

**Figure 7:**
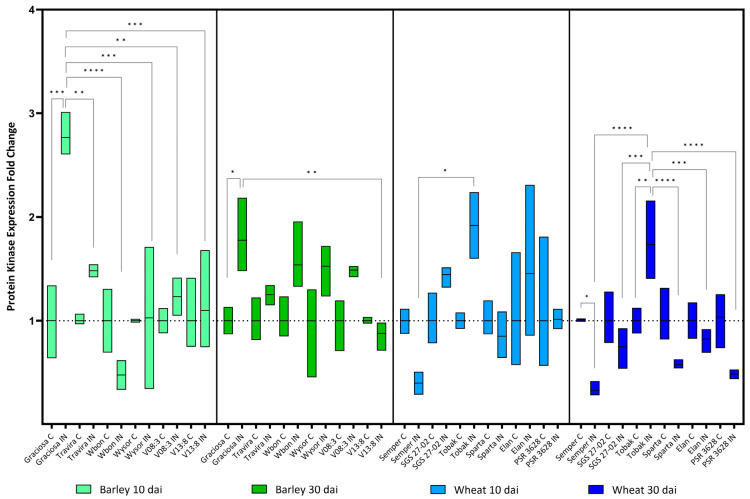
Box plots showing the expression fold change of *Protein kinase* in different barley and wheat genotypes at 10 and 30 dai. The horizontal line in each box represents the mean expression fold change, and the lower and upper box limits the minimum and maximum values, respectively. Two-way ANOVA and Tukey’s multiple comparison test revealed significant differences * = *p* < 0.05, ** = *p* < 0.01, *** = *p* < 0.001, and **** = *p* < 0.0001.

*Protein kinase* expression in barley was increased at 10 dai exclusively in the susceptible genotype Graciosa, but by 30 dai, expression had decreased and remained significant compared to the control and infected V13:8 (Figure 7). In wheat, *Protein kinase* gene expression was affected only slightly. According to Tukey’s test, only the infected Semper differed significantly from infected Tobak. However, at 30 dai the difference is notable, mainly due to significantly decreased expression in Semper as opposed to increased expression in Tobak; this increased expression was also significant compared to the other genotypes and lines (Barley 10 dai BYDV-PAV -genotype interaction F (5, 21) = 7.35, *p* = 0.0004; genotype effect *p* = 0.0004; and BYDV-PAV effect *p* = 0.008), (barley 30 dai BYDV-PAV-genotype interaction F (5, 24) = 2.26, *p* = 0.081; genotype effect *p* = 0.081; and BYDV-PAV effect *p* < 0.0001), (wheat 10 dai BYDV-PAV-genotype interaction F (5, 23) = 2.68, *p* = 0.047; genotype effect *p* = 0.047; and BYDV-PAV effect *p* = 0.19) and (Wheat 30 dai BYDV-PAV-genotype interaction F (5, 24) = 9.81, *p* < 0.0001; genotype effect *p* < 0.0001; and BYDV-PAV effect *p* = 0.002) according to two-way ANOVA with Tukey’s multiple comparison test).

**Figure 8:**
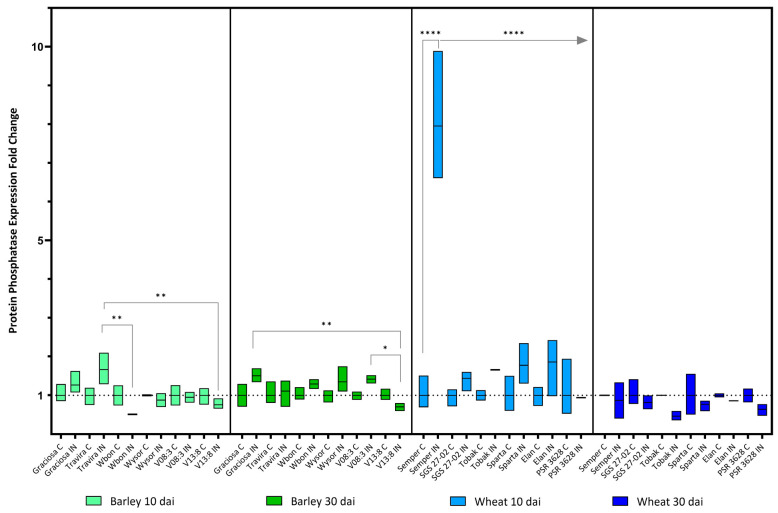
Box plots showing the expression fold change of Protein phosphatase in different barley and wheat genotypes at 10 and 30 dai. The horizontal line in each box represents the mean expression fold change, and the lower and upper limits the minimum and maximum values, respectively. Two-way ANOVA and Tukey’s multiple comparison test revealed significant differences * = *p* < 0.05 ** = *p* < 0.01 and **** = *p* < 0.0001.

Some significant differences in *Protein phosphatase* gene expression were found between the infected barley genotypes, at 10 dai between Travira vs. Wbon and Travira vs. V13:8, and at 30 dai between Graciosa vs. V13:8 and V08:3 vs. V13:8 (Figure 8). As in wheat, the expression of the gene *Protein phosphatase* was significantly increased in the infected Semper samples, but only at 10 dai. (According to two-way ANOVA with Tukey’s test: (barley 10 dai BYDV-PAV -genotype interaction F (5, 21) = 7.35, *p* = 0.0004; genotype effect *p* = 0.0004; and BYDV-PAV effect *p* = 0.008), (barley 30 dai BYDV-PAV -genotype interaction F (5, 24) = 2.26, *p* = 0.081; genotype effect *p* = 0.081; and BYDV-PAV effect *p* < 0.0001), (wheat 10dai BYDV-PAV -genotype interaction F (5, 23) = 2.68, *p* = 0.047; genotype effect *p* = 0.047; and BYDV-PAV effect *p* = 0.19) and (wheat 30 dai BYDV-PAV -genotype interaction F (5, 24) = 9.81, *p* < 0.0001; genotype effect *p* < 0.0001; and BYDV-PAV effect *p* = 0.002).

**Figure 9:**
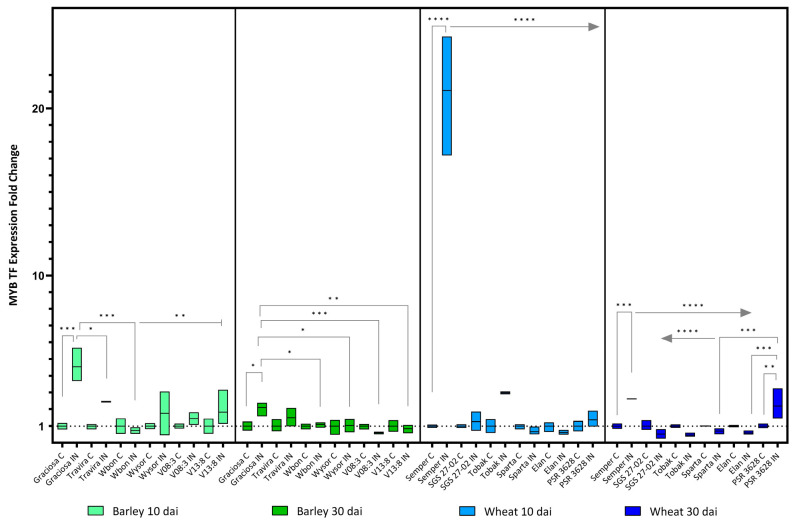
Box plots showing the expression fold change of *MYB TF* in different barley wheat genotypes at 10 and 30 dai. The horizontal line in each box represents the mean expression fold change, and the lower and upper box limits the minimum and maximum values, respectively. Two-way ANOVA and Tukey’s multiple comparison test revealed significant differences * = *p* < 0.05, ** = *p* < 0.01, *** = *p* < 0.001, and **** = *p* < 0.0001.

The *MYB transcription factor* (TF), i.e., GAMYB for barley, was up-regulated in Graciosa in response to BYDV-PAV infection at 10 dai and less markedly at 30 dai. Expression of this transcription factor in wheat, RIM1, was also strongly increased at 10 dai in the susceptible genotype Semper and remained moderately increased at 30 dai, at which time, however, an opposite effect was observed; for example, a similarly significant increase in the resistant line PSR 3628 (Figure 9). ANOVA with Tukey’s test: (barley 10dai BYDV -genotype interaction F (5, 21) = 5.31, *p* = 0.003; genotype effect *p* = 0.003; and BYDV effect *p* = 0.0001), (barley 30 dai BYDV-PAV -genotype interaction F (5, 24) = 3.93, *p* = 0.01; genotype effect *p* = 0.01; and BYDV-PAV effect *p* = 0.08), (wheat 10 dai BYDV-PAV -genotype interaction F (5, 23) = 80.46, *p* < 0.0001; genotype effect *p* < 0.0001; and BYDV-PAV effect *p* < 0.0001) and (wheat 30 dai BYDV-PAV -genotype interaction F (5, 24) = 12.39, *p* < 0.0001; genotype effect *p* < 0.0001; and BYDV-PAV effect *p* = 0.096)

**Figure 10:**
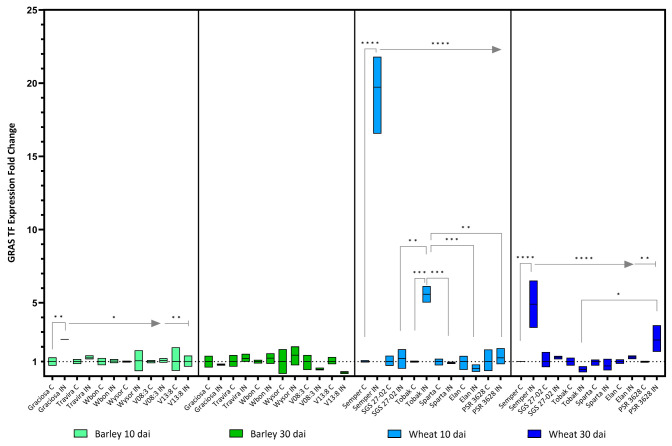
Box plots showing the expression fold change of *GRAS TF* in different barley and wheat genotypes at 10 and 30 dai. The horizontal line in each box represents the mean expression fold change, and the lower and upper box limits the minimum and maximum values, respectively. Two-way ANOVA and Tukey’s multiple comparison test revealed significant differences * = *p* < 0.05, ** = *p* < 0.01, *** = *p* < 0.001, and **** = *p* < 0.0001.

The expression of the *GRAS transcription factor (Gras)* in barley was increased only in Graciosa at the beginning of infection, but no significant changes were detected at 30 dai. Expression of *Gras* in wheat genotypes was dramatically increased 10 dai in the infected Semper and moderately increased in the infected Tobak genotype, but at 30 dai, expression levels decreased back to normal (Figure 10). Two-way ANOVA with Tukey’s test result: (barley 10 dai BYDV-PAV -genotype interaction F (5, 21) = 3.26, *p* = 0.025; genotype effect *p* = 0.025; and BYDV-PAV effect *p* = 0.03), (barley 30 dai BYDV-PAV -genotype interaction F (5, 24) = 1.92, *p* = 0.13 ns; genotype effect *p* = 0.13 ns; and BYDV-PAV effect *p* = 0.49 ns), (wheat 10dai BYDV-PAV -genotype interaction F (5, 23) = 97.78, *p* < 0.0001; genotype effect *p* < 0.0001; and BYDV-PAV effect *p* < 0.0001) and (wheat 30 dai BYDV-PAV -genotype interaction F (5, 24) = 9.59, *p* < 0.0001; genotype effect *p* < 0.0001; and BYDV-PAV effect *p* = 0.0003).

**Figure 11:**
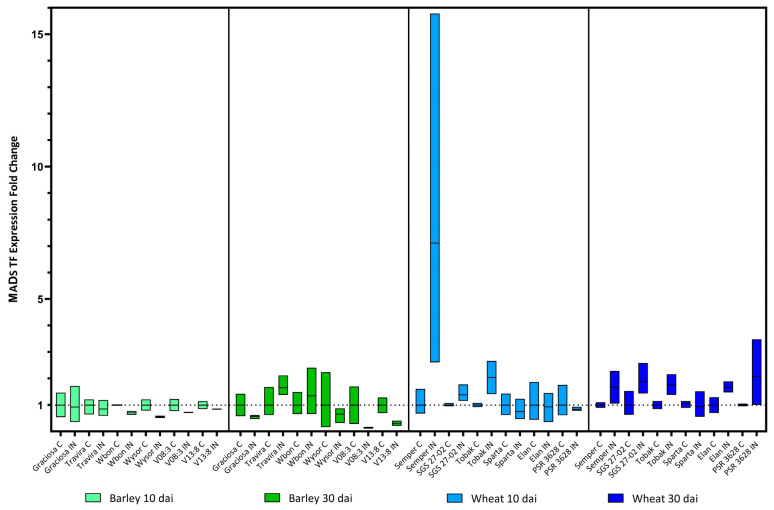
Box plots showing the expression fold change of MADS box transcription factor in different barley and wheat genotypes at 10 and 30 dai. The horizontal line in each box represents the mean expression fold change, and the lower and upper box limits the minimum and maximum values, respectively. Two-way ANOVA and Tukey’s multiple comparison test did not revealed any significant differences.

The straight line from on top indicates the same *p* value when that genotype is compared to the rest in line. The *MADS box TF* was the only gene for which no significant change in expression was found, either in barley or wheat genotypes (Figure 11). In agreement with the two-way ANOVA and Tukey’s test: (barley 10 dai BYDV-PAV-genotype interaction F (5, 21) = 0.25, *p* = 0.93 ns; genotype effect *p* = 0.93 ns; and BYDV-PAV effect *p* = 0.05), (barley 30 dai BYDV-PAV-genotype interaction F (5, 24) = 1.77, *p* = 0.16 ns; genotype effect *p* = 0.16 ns; and BYDV-PAV effect *p* = 0.24 ns), (wheat 10 dai BYDV-PAV -genotype interaction F (5, 23) = 1.78, *p* = 0.16 ns; genotype effect *p* = 0.16 ns; and BYDV-PAV effect *p* = 0.14 ns) and (wheat 30 dai BYDV-PAV -genotype interaction F (5, 23) = 0.90, *p* = 0.50 ns; genotype effect *p* = 0.61 ns; and BYDV-PAV effect *p* = 0.0013).

### 3.3. Comparison of Gene Expression Profiles of Highly Resistant versus Highly Susceptible Genotypes of Wheat and Barley

To demonstrate gene expression and resistance of wheat and barley genotypes, we presented here the comparison of gene expression fold changes (EFC) between the highly susceptible genotypes Semper (wheat) and Graciosa (barley) and the highly resistant genotypes PSR 3628 (wheat) and Wysor (barley) (Table 1). A paired sample T-test was performed to compare the infected vs. control and the susceptible vs. resistant. Statistically significant changes were observed in wheat in the up-regulation of *NBS*, *CC-NBS-LRR* and *RLK* in the susceptible genotype and down-regulation in the resistant genotype in response to BYDV-PAV. Similar up-regulation of *NBS*, *CC-NBS-LRR*, *RLK* and *MYB TF* was also observed in the susceptible barley genotype. However, in the resistant barley genotype, no change in expression was detected except for the down-regulation of *RLK*. *Casein kinase* and *Protein phosphatase* were up-regulated in the susceptible genotype of wheat, and only in the early dai, but in the later dai, *Protein phosphatase* is down-regulated in the resistant genotype of wheat. *Protein kinase* was down-regulated both earlier (10 dai) and later (30 dai) in the susceptible wheat genotypes, but only in the later dai in the resistant genotypes. *GRAS TF* and *MYB TF* were up-regulated in the susceptible wheat genotype and there were no significant differences in *MADS TF* expression. *Protein kinase*, *Casein kinase* (30 dai), *MYB TF* and *GRAS TF* (10 dai) were up-regulated in the susceptible barley genotype. No significant differences were found in the expression of *Protein phosphatase* and *MADS FT* genes between the barley genotypes (Table 1). Similar results were found in other genotypes studied with respect to the levels of resistance or susceptibility to BYDV-PAV (Supplementary Table S2). Our results showed a clear differentiation of expression patterns related to resistance and susceptibility in both wheat and barley genotypes. Therefore, *RLK*, *NBS*, *CC-NBS-LRR*, *GRAS TF* and *MYB TF* could be potential markers for BYDV-PAV resistance breeding and targets for cereal crop improvement. In particular, the *RLK* gene, which showed the same expression pattern in both wheat and barley genotypes and specific to resistance or susceptibility, is of note.

## 4. Discussion

We have studied the gene expression profiles of wheat and barley genotypes to identify novel genes associated with resistance to BYDV-PAV. While some resistance (or tolerance) genes are known to confer resistance (e.g., *BYd2* gene) (reviewed in [16]), their introduction into wheat or barley varieties remains limited. Based on our previous transcriptome analysis of miRNAs and their target genes [23], as well as known genes associated with disease resistance to several other pathogens [18,30], the expression profile of several NBS family target genes (e.g., *NBS-LRR, CC-NBS-LRR*), kinases (e.g., *Casein kinase, Protein kinase*, *Protein phosphatase*), receptor-like kinases (*LRR-RLKs)* and transcription factors (e.g., *MYB, GRAS*, *MADS*) was analysed for their possible role in BYDV-PAV resistance. In general, gene expression profiles together with virus titre analyses showed that infection of BYDV-PAV causes significant changes in gene expression depending on genotype resistance and time after virus inoculation. In particular, our results showed that *NBS, CC-NBS-LRR, RLK, Protein kinase*, *MYB TF*, *GRAS TF* were upregulated in susceptible genotypes only at 10 dai and *Casein kinase* 30 dai, and *RLK* was down-regulated in resistant barley genotypes (only 30 dai). Similarly, in wheat *NBS*, *CC-NBS-LRR*, *RLK*, *MYB TF*, *GRAS TF*, *Protein phosphatase*, and *Casein kinase* were upregulated only at 10 dai, in susceptible genotypes, and *RLK* and *Protein kinase* were down-regulated 10 dai in resistant genotypes.

### 4.1. NBS-LRR Family Genes Trigger BYDV-PAV Defense Response in Susceptible Genotypes

Two of the selected genes, NBS and CC-NBS-LRR, belong to the largest family of R genes, which encode proteins characterised by a structurally conserved region, the NBS, usually associated with a leucine-rich repeat LRR [33]. This family can be subdivided based on characteristic N-terminal features of its products [34], such as a CC that enables protein–protein interactions. In our analysis, NBS and CC-NBS-LRR were expressed at relatively low levels in uninfected leaves. However, they were remarkably up-regulated in susceptible genotypes at 10 dai, and the response persisted at least until 30 dai. Increased expression of NBS-LRR genes in response to pathogen infection has also been observed in other plants, e.g., AhRRS5, in peanut in response to Ralstonia solanacearum [35]; Xa1, a bacterial resistance gene from rice [36]; SacMi, triggered by infection with Meloidogyne incognita [33]; and ZmNBS25, associated with Bipolaris maydis resistance in maize [37]. In a report on Vitis vinifera infected with Erysi-phenecator (the causal agent of powdery mildew), 63 powdery mildew-responsive NBS-LRR genes were identified whose expression levels differed between susceptible and partially resistant genotypes and at different time points (1-5 dai). This led to the conclusion that NBS-LRR genes play an important role in activating defence mechanisms as powdery mildew infection progresses and that their expression is conserved in the grape genotypes [38]. In our analysis, the up-regulation of NBS and CC-NBS-LRR in the susceptible genotypes is associated with a significant increase in BYDV-PAV copy number, which might have activated the defence mechanisms early upon BYDV-PAV infection but continued until 30 dai. The fact that expression is maintained at baseline levels in the resistant genotypes could be a consequence of alternative resistance mechanisms that inhibited viral replication (lower BYDV-PAV titre) possibly triggered early in BYDV-PAV infection. In this regard, we are only dissecting part of a complicated network that needs to be further explored in more detail. Previous studies report the completion of host virulence targets with “integrated NBS pairs” (integrated Decoy hypothesis) that function together to confer resistance, for example the rice CC-NBS -LRRs RGA4 and RGA5 that recognise the Magnaporthae oryzae effectors Avr-Pia and Avr-CO39 [39]; barley CC-NBS -LRRs HvRga1 and Rpg5 mediating perception of the stem rust pathogen Puccinia graminis [40,41,42,43,44]; and the wheat pair Lr10 and RGA2 mediating resistance to P. triticina [45]. In particular, the miRNA analysis of Jarošová et al. [23] helped to limit our selection to conserved NBS-LRR gene expression in response to BYDV-PAV, considering that in barley, over 400 NBS-LRR genes have been identified by genome-wide analysis, while hexaploid wheat T. aestivum has over 2000 NBS-LRR genes due to polyploidisation. Only a fraction of the sublines is retained in both T. aestivum and H. vulgare, reflecting a rapid change in NBS-LRR profiles after separation of the species due to species-specific gene loss and duplication [46]. The similarity of the expression profiles of six genotypes each of barley and six wheat suggests that the expression of the selected NBS and CC-NBS-LRR genes are conserved in the genotypes included in our study and also between the two species.

### 4.2. Casein Kinase, Protein Kinase and Protein Phosphatase as Integrated Domains after BYDV-PAV Infection

In addition to the integrated *NBS* pairs found in barley and wheat [37,38,39,40,41], unusual domains have been identified in the standard functional *NBS-LRR* protein domain architectures required for activation of pathogen-induced defence signalling. It has been proposed that these accessory domains be termed ‘Integrated Sensory Domains’ (ISDs) until their function is elucidated [47]. The NBS ISDs allow for functional diversity, as the integration of different ISDs into a conserved *NBS-LRR* pair enables defence against a broader range of pathogens and subsequently initiates the defence response. In barley, the alleles of Rpg5 contain functionally different C-terminal ISDs. Resistance alleles have a serine-threonine protein kinase ISD, while the major class of susceptible alleles of Rpg5 contains a protein phosphatase 2C ISD [48]. Jarošová et al. [23] identified novel miRNA in barley genotypes infected with BYDV-PAV that could express ISDs; from these we selected *Casein kinase*, *Protein kinase* and *Protein phosphatase* domains for this study. Our results showed different expression patterns between barley and wheat, suggesting that the composite NBS ISDs that respond to BYDV-PAV infection may be species specific. For *Casein kinase*, a distinct up-regulation was detected in susceptible genotypes, but this was not detected until 30 dai in barley and 10 dai in wheat. For the *Protein kinase* domain, expression is up-regulated at 10 dai in the susceptible barley genotype Graciosa, but the effect is almost lost by day 30. In contrast, the susceptible wheat genotype Semper is down-regulated at this time, while the winter-tolerant genotype Tobak is up-regulated. As far as *Protein Phosphatase* expression is concerned, despite the differences between some barley genotypes, there is an obvious effect of BYDV-PAV infection, while in the susceptible wheat genotype Semper, there is a remarkable up-regulation by BYDV-PAV, but this does not last until day 30. To draw a general conclusion about the expression of ISDs in response to BYDV-PAV infection, a more detailed study is needed, following the trail at multiple time points and including other proteins involved in the signalling pathway.

### 4.3. Receptor like Kinase Gene Defense Response to BYDV-PAV

Many *receptor-like kinases (RLK)* in plants have been shown to perceive and process signals from invading pathogens. They serve as pattern recognition receptors (PRRs) that are rapidly activated by specific pathogen effectors. *LRR-RLKs* play an important role in the trade-off between growth and immunity. In the plant immune response, they trigger specific phosphorylation events inside and outside the kinase domain, leading to altered kinase activity and thus to the transmission of immune signals [49,50]. To date, the function of multiple *RLK*s in pathogen resistance has been extensively studied in model plants with simpler genomes. In contrast, much less is known about the role of *RLK*s in disease resistance in crops with complex genomes such as wheat and barley [51]. Choudhury et al. [9] identified specific genomic regions for BYDV-PAV resistance in wheat genotypes through a genome-wide association study. Between the annotated genes was a kinase-like receptor gene that mediated disease resistance by activating the cellular defence response and an LRR receptor kinase that functioned as a basal defence against *Fusarium* head blight and as an upstream component of salicylic acid signalling [52]. Thapa et al. [53] analysed *Poaceae*-specific *LRR-RLKs*, especially homologues between *H. vulgare* and *T. aestivum*, and found by gene expression studies that TaLRRK-6D is systemically activated by *F. graminearum* as early as 1 dai in the susceptible wheat genotype Remus and that the expression of TaLRRK-6D was always higher compared to the resistant genotype CM82036. Their virus-induced gene silencing analysis suggests that TaLRRK-6D in wheat and its barley homologue HvLRRK-6H contributed positively to *F. graminearum* resistance. In response to barley PM resistance, contrasting genotype-specific expression patterns were observed in TaRLK-R1, -R2 and -R2, HvXa21RLK and other closely related *RLK*s, suggesting that specific *RLK*s are differentially important for specific Mla-mediated resistance [54,55]. Parrot et al. [56] identified three other (putative) *LRR-RLKs* that show an inverse pattern in response to the Mla locus (low in the presence of Mla1 and Mla13, but high in the presence of Mla6), while other putative PRRs show low expression in the presence of Mla13, but high expression in the presence of both Mla1 and Mla6. In this regard, to our knowledge, there is no report of viral defence response in genotypes with resistance mediated by *Ryd*1, *Ryd*2 or *Ryd*3 in barley or by *Bvd2* in wheat genotypes and their interaction with *RLK*s. The barley and wheat *RLK genes (Rec Kin)* examined in this study were up-regulated 10 days after BYDV-PAV infection only in the susceptible genotypes Graciosa (barley), Semper and SGS 27-02 (wheat) in parallel with a higher number of virus copies. In contrast, the resistant wheat genotypes Sparta and PSR 3628 showed down-regulation compared to their respective controls. The up-regulation in Graciosa and Semper continued until day 30 post infection. At this time, the Vir8:13 and Vir13:8 lines with pyramidal resistance genes showed contrasting down-regulation. The clear difference between susceptible and resistant genotypes in the expression of the selected *LRR-RLK* genes underlines their involvement in BYDV-PAV defence and the relevance of further studies of these genes for the implementation of resistant genotypes *(*e.g., introduction into new genotypes, gene silencing, pathway analysis, interaction with other *RLK*, etc.).

### 4.4. Expression of MYB, GRAS and MADS TFs in Response to BYDV-PAV Infection

Transcription factors are key components in plant adaptation mechanisms and defence signals. Therefore, several studies have functionally characterized TF genes in different plant species to improve their resistance/tolerance to different stresses, especially for crop improvement [57]. In this study, we included wheat and barley genes encoding *MYB*, *GRAS* and *MADS-box TFs*. Plants contain TFs characterized by a DNA-binding *MYB* domain. Based on the number of adjacent MYB repeats, *MYB TFs* can be classified into four classes, with most of the identified *MYB* genes in plants belonging to the R2R3 *MYB* subfamily and associated with plant defence responses [58]. For example, AtMYB96 in Arabidopsis may increase resistance to bacterial pathogens [59], AtMYB59 plays a role in hormonal signalling pathways in response to biotic stress [60] and BOS1 (AtMYB108) is required to limit the spread of two necrotrophic pathogens, *Botrytis cinerea* and *Alternaria brassicicola* [61]. In barley, the *MYB TF* HvMYB6 functions as a positive regulator of basal and MLA-mediated immunity responses against *Blumeria graminis* [62]. Similarly, several R2R3-type *MYB TFs* have been shown to play a role in defence against pathogens in wheat. For example, silencing of the TaMYB4 gene in wheat impaired resistance to *Puccinia striiformis*, while TaPIMP1-expressing plants showed significantly increased resistance to *R. solanacearum* in transgenic tobacco [63] and resistance to *Bipolaris sorokiniana* in transgenic wheat [64]; the TaRIM1 gene was induced by infection with *R. cerealis* and positively contributed to the resistance response of wheat by regulating the expression of several defence-related genes [57]. In our analysis, *MYB TF* was up-regulated in the susceptible genotypes Graciosa and Semper in response to BYDV-PAV infection at 10 dai, and the up-regulation remains less pronounced at 30 dai. Nevertheless, a slight up-regulation was also detected at this time in the wheat resistance genotype PSR 3628. The *MYB* gene we identified for wheat is up-regulated in genotypes with opposite resistance effects, for which we can only assume that the conferred resistance is orchestrated with different players along the way. For example, efficient expression and function of an *RLK* in wheat powdery mildew defence requires a *MYB TF* binding site located in the intron [52].

The *GRAS TFs* family is found throughout the plant kingdom. They perform numerous biological functions in plants, including growth, development, cell signalling, phytochrome signalling, symbiosis, biotic and abiotic stress tolerance, etc. [65,66,67,68]. To et al. [69] identified 62 barley GRAS proteins, while for wheat 183 *GRAS* genes were found coding for 194 GRAS proteins [70]. In response to BYDV-PAV infection, our data suggest an up-regulation of *GRAS TF* in the susceptible barley genotype Graciosa only at day 10 post-infection, while in wheat a remarkable up-regulation was shown for the susceptible genotype Semper and to a lesser extent for Tobak, but later, 30 dai, only a slightly increased expression for Semper remained. A more detailed study of the sequence we have targeted should follow to clarify whether the effect as DELLA proteins (members of the *GRAS TF* family) is related to proteins previously described in Arabidopsis, rice and maize as major growth inhibitors responsible for dwarfism and key components in gibberellic acid signalling [71]. Rong et al. [72] reported up-regulation of gibberellic acid receptor GID1L2 and gibberellic acid-stimulated transcripts and down-regulated transcript encoding gibberellin receptor GID1L3 for the susceptible wheat line Zhong8601, 35 dai of BYDV-GAV infection, suggesting that altered expression of these genes in these phytohormone pathway plants could lead to plant dwarfism. In this respect, our data may suggest that BYDV-PAV infection affects signalling at the *TF* level in susceptible genotypes.

The *MADS box transcription factor* family is an important selection target in crop domestication and improvement, as they are key regulators of virtually every aspect of plant reproductive development [73]. Recent findings also link *MADS-box* genes to disease resistance, e.g., MADS1 regulates the defence response of tobacco to HarpinXoo infections [74], down-regulation of OsMADS26 in transgenic rice increased resistance to rice blast and bacterial wilt [75], OsRDR1 expression is a key component of the antiviral RNA pathway and increased resistance to rice stripe virus (RSV) [76], and GmCAL was significantly induced after inoculation with soybean mosaic virus (SMV) and its overexpression significantly reduced SMV accumulation [77]. However, for the specific times of our analysis, *MADS TF* was the only target gene for which no significant change in expression was detected after BYDV-PAV, neither in barley nor in wheat genotypes.

## 5. Conclusions

In wheat and barley, the gene expression profile together with the virus titre analyses revealed that BYDV-PAV infection causes significant changes in eight of the selected genes, depending on both the level of genotype resistance and the post-infection time point. The following conclusions can be drawn from our results: (i) gene expression, together with virus titre analysis, can be used for determining the resistance levels of wheat and barley to BYDV-PAV; (ii) BYDV-PAV infection has little or no effect on the expression of most of the listed genes in resistant wheat and barley genotypes, while they are mostly upregulated in susceptible genotypes; (iii) the *NBS, CC-NBS-LRR* and *RLK* genes showed unique expression patterns in response to BYDV-PAV in wheat and barley genotypes, which equally differ in their resistance or susceptibility to the virus and mediate a defence response; (iv) the transcription factors *GRAS* in wheat and *MYB* in barley also showed a similar expression pattern and may also contribute to the defence response in wheat and barley, respectively; (v) the *RLK* gene showed the same expression pattern associated with resistance or susceptibility in both wheat and barley genotypes, indicating a unique candidate gene, conferring resistance.

These target genes thus provide a new avenue for further investigation of the molecular and physiological processes involved in the defence of barley and wheat against BYDV-PAV. They could be particularly useful for monitoring and developing BYDV-PAV-resistant cereal plants using molecular breeding methods, including transgenic breeding and marker-assisted selection of elite alleles. In this sense, finding orthologous resistant genotypes will increase the possibility of selecting superior alleles for breeding. However, additional factors should be taken into account, such as genetic adaptations of barley (a diploid with a genome size of 5.1 Gb) and wheat (a hexaploid with a genome size of 16 Gb) species, different gene expression levels between genotypes, early or late response to infection and interdependence between effector genes or their products in the signalling pathways they trigger.

## Figures and Tables

**Table 1:** Gene expression in highly resistant versus highly susceptible genotypes of wheat and barley. The values include mean of the expression fold change (EFC), standard error of mean (SEM) and T-test *p* values. EFC above 1 shows up-regulation (blue) of the gene relative to the control; values below 1 are indicative of gene down-regulation (green) relative to the control. Yellow highlights *p* < 0.05.

			Susceptible Wheat Genotype	Resistance Wheat Genotype			Susceptible Barley Genotype	Resistance Barley Genotype	
Target Gene	DAI		Semper Control vs. Infected EFC	PSR 3628 Control vs. Infected EFC	Semper In vs. PSR 3628 In		Graciosa Control vs. Infected EFC	Wysor Control vs. Infected EFC	Graciosa In vs. Wysor In
*NBS-LRR*	10 dai	mean	31.864	1.887		mean	3.843	0.874	
		SEM	2.684	0.491		SEM	0.734	0.173	
		TTEST	0.0003	0.251	0.0013	TTEST	0.020	0.635	0.050
	30 dai	mean	30.701	0.310		mean	3.314	1.208	
		SEM	8.088	0.034		SEM	0.835	0.222	
		TTEST	0.016	0.043	0.0153	TTEST	0.050	0.450	0.0714
*CC-NBS-LRR*	10 dai	mean	13.875	0.765		mean	5.845	0.857	
		SEM	2.846	0.213		SEM	0.691	0.190	
		TTEST	0.012	0.757	0.0508	TTEST	0.002	0.561	0.012
	30 dai	mean	5.949	0.443		mean	2.857	1.439	
		SEM	0.899	0.030		SEM	0.324	0.103	
		TTEST	0.005	0.013	0.0049	TTEST	0.007	0.044	0.0140
*Receptor-like Kinase*	10 dai	mean	20.345	0.396		mean	7.021	0.472	
		SEM	2.165	0.042		SEM	2.019	0.129	
		TTEST	0.001	0.044	0.0008	TTEST	0.041	0.050	0.087
	30 dai	mean	9.030	1.152		mean	10.429	0.402	
		SEM	0.868	0.282		SEM	0.964	0.122	
		TTEST	0.001	0.650	0.0006	TTEST	0.0006	0.038	0.0005
*Casein Kinase*	10 dai	mean	6.161	0.949		mean	1.723	1.158	
		SEM	1.907	0.196		SEM	0.277	0.269	
		TTEST	0.054	0.927	0.0776	TTEST	0.106	0.589	0.676
	30 dai	mean	0.905	0.962		mean	2.621	1.129	
		SEM	0.158	0.262		SEM	0.179	0.072	
		TTEST	0.550	0.901	0.3249	TTEST	0.001	0.546	0.0015
*Protein Phosphatase*	10 dai	mean	7.951	0.941		mean	1.268	0.877	
		SEM	0.992	0.001		SEM	0.180	0.177	
		TTEST	0.002	0.906	0.0040	TTEST	0.309	0.424	0.239
	30 dai	mean	0.868	0.639		mean	1.504	1.348	
		SEM	0.467	0.088		SEM	0.103	0.201	
		TTEST	0.729	0.055	0.8054	TTEST	0.063	0.191	0.5278
*Protein Kinase*	10 dai	mean	0.397	1.012		mean	2.769	1.027	
		SEM	0.063	0.056		SEM	0.124	0.685	
		TTEST	0.003	0.978	0.0354	TTEST	0.002	0.962	0.047
	30 dai	mean	0.326	0.484		mean	1.775	1.524	
		SEM	0.045	0.027		SEM	0.212	0.148	
		TTEST	0.0001	0.025	0.0085	TTEST	0.026	0.168	0.3862
*MYB TF*	10 dai	mean	21.074	1.361		mean	4.540	0.549	
		SEM	2.078	0.282		SEM	0.592	0.041	
		TTEST	0.001	0.338	0.0006	TTEST	0.004	0.066	0.110
	30 dai	mean	2.617	2.195		mean	2.106	1.038	
		SEM	0.035	0.539		SEM	0.261	0.222	
		TTEST	0.001	0.093	0.0007	TTEST	0.022	0.917	0.0355
*GRAS TF*	10 dai	mean	19.726	1.258		mean	2.510	1.062	
		SEM	1.612	0.333		SEM	0.013	0.710	
		TTEST	0.0003	0.663	0.0003	TTEST	0.001	0.914	0.072
	30 dai	mean	4.910	2.463		mean	0.786	1.437	
		SEM	1.614	0.530		SEM	0.039	0.371	
		TTEST	0.047	0.051	0.0430	TTEST	0.408	0.514	0.1557
*MADS TF*	10 dai	mean	7.112	0.844		mean	4.540	1.764	
		SEM	4.337	0.046		SEM	0.592	1.296	
		TTEST	0.233	0.702	0.2169	TTEST	0.888	0.490	0.524
	30 dai	mean	1.674	2.064		mean	0.569	0.658	
		SEM	0.617	0.735		SEM	0.047	0.169	
		TTEST	0.244	0.221	0.3046	TTEST	0.157	0.629	0.6372

## Data Availability

Not applicable.

## Supplementary Materials

The following supporting information can be downloaded at: https://www.mdpi.com/article/10.3390/v15030716/s1, Supplementary Table S1. List of primers used in this study; Supplementary Table S2. Expression profile showing the expression fold change mean values, SEM and TTest; Supplementary Figure S1. BYDV-PAV titre for all barley and wheat samples with multifactorial ANOVA values.

## Abbreviations

BYDV-PAV	Barley yellow dwarf virus-PAV
RT-qPCR	Reverse transcription-quantitative polymerase chain reaction
miRNAs	Micro-Ribonucleic acid
PGSB	Plant Genome and Systems Biology
LRR	Leucine-rich repeat
R	Resistance (used as R gene or R protein)
Dai	Days after inoculation
Ct	Cycle threshold
NCBI	National Center for Biotechnology Information
E	Efficiency (primer)
ANOVA	Analysis of variance
NBS	Nucleotide Binding Site
LRR	Leucine-rich repeat
CC	Coiled coil
TF	Transcription factor
ISD	Integrated sensory domains
RLK	Receptor-like kinases
PRP	Pattern-recognizing receptor
MYB	Myeloblastosis (domain of the transcription factor)
GRAS	Transcription factor named after the first three members: gibberellic-acid insensitive (GAI), repressor of GAI (RGA) and Scarecrow (SCR)
MADS	Acronym is derived from the yeast Minichromosome Maintenance 1 (MCM1), the Arabidopsis agamous (AG), the Antirrhinum deficiens (DEFA), and the mammalian Serum Response Factor (SRF)
