# Supplementary material for: Identifying Putative Resistance Genes for Barley Yellow Dwarf Virus-PAV in Wheat and Barley
Source: Viruses. 2023 Mar 9;15(3):716. doi: 10.3390/v15030716 (PMC10053949; doi:10.3390/v15030716)
Supplement: Supplementary file 1 [file viruses-15-00716-s001.zip › Supplementary Table S1-S2-Figure S1_Revised.pdf]

viruses

MDPI

# Title: Identifying Putative Resistance Genes for Barley Yellow Dwarf Virus-PAV in Wheat and Barley

**Table S1.** List of the primers used in this study.
(sequences, amplicon sizes, GenBank (NCBI database) accession numbers and efficiencies of all primers used in qPCR experiments with samples of *Hordeum vulgare* and *Triticum aestivum* (2 pairs designed per gene of interest). Primers for reference genes and BYDV-PAV standard [17] are also included.)

| **ID** | **Target Gene** | GenBank | species | Sequence (5'->3') | | position | Amplicon size (bp) | Efficiency (%) | References |
|---|---|---|---|---|---|---|---|---|---|
| NBS | rga S-9203: resistance gene of the NBS-LRR class | AJ507095.1 | *Hordeum vulgare* | 1F | CACCACCGAGAGAATAGAACCA | 17-38 | 170 | 110 | |
| | | | | 1R | TCCTTGAGAAACGCCACCT | 186-168 | | | |
| | | | | 2F | TGTTGGGTTTGGGGGTCT | 652-669 | 172 | 110 | |
| | | | | 2R | GTCAGTATGGGTTGCTTGTGG | 823-803 | | | |
| | NBS-LRR resistance gene homologue from TcLr35 | DQ205351.2 | *Triticum aestivum* | 1F | TGGTCGGTTTTGAGGACGAG | 726-745 | 113 | 94 | |
| | | | | 1R | AAGCGTTGTTTTTCCTGCCC | 838-819 | | | |
| | | | | 2F | AGGGTTGCAGTCTTGGTGAG | 484-423 | 150 | 103 | |
| | | | | 2R | CAAGTCAGTCGGTAGGCGAG | 553-534 | | | |
| CC-NBS-LRR | disease resistance protein RGA2-like | AK371901.1 | *Hordeum vulgare* | 1F | GCAGTGTTTCCGGGTGACTA | 1539-1558 | 145 | 105 | |
| | | | | 1R | AGCATCGCTCGACAAGTTCA | 1683-1664 | | | |
| | | | | 2F | ACGCCAAATGGTACAGCTCA | 2321-2340 | 71 | 110 | |
| | | | | 2R | TGAACCCACTAGCATCGCTC | 2391-2372 | | | |
| | coiled-coil nucleotide-binding leucine-rich | MW656173.1 | *Triticum aestivum* | 1F | CCACCTCCTCTCGTACTCCA | 11402-11-421 | 85 | 106 | |

| | | | | | | | | | |
|---|---|---|---|---|---|---|---|---|---|
| | repeat (CC-NB-LRR) immune receptor (Lr13) gene | | | 1R | ACTGGTATGCTGTTCTGCCC | 11486-11467 | | | In this study |
| | | | | 2F | TTGCTGGCGTGATTGACTCT | 17656-17675 | 163 | 93 | |
| | | | | 2R | GTTGAAGGTAGGGTGCCGAA | 17818-17799 | | | |
| Rec Kin | LRR receptor-like serine/threonine-protein kinase At3g47570 | AK364948.1 | *Hordeum vulgare* | 1F | TTTACTGCGGTGGGAGCATT | 632-651 | 88 | 108 | |
| | | | | 1R | CTTGGTGGGCATCACCTCTT | 719-700 | | | |
| | | | | 2F | TGTTCGCTGCTCCAGTTTCA | 1569-1588 | 89 | 110 | |
| | | | | 2R | TTCTTTCACCACATCGCCCA | 1657-1638 | | | |
| | LRR receptor-like kinase | GU084176.1 | *Triticum aestivum* | 1F | GCTTCTTCTGCTGGCGTTTC | 125-144 | 154 | 98 | |
| | | | | 1R | GCTTCGGTTCCAGTTCCTCA | 278-259 | | | |
| | | | | 2F | TAACGACAGCGAAAACCCCA | 2642-2661 | 95 | 107 | |
| | | | | 2R | TGCCGAAATCCCCCAAATGA | 2736-2717 | | | |
| Cas Kin | Casein kinase | MT363976.1 | *Hordeum vulgare* | 1F | ACTACAAAGGCCGTCAAGGG | 617-636 | 71 | 101 | |
| | | | | 1R | AACATCCCAAAGGCTAGGGC | 687-668 | | | |
| | | | | 2F | AAGATGGATGGCGTGAGTCC | 313-332 | 148 | 102 | |
| | | | | 2R | TGCCAACATAAACCTGCCCA | 460-441 | | | |
| | Casein kinase-like protein | AF479055.1 | *Triticum aestivum* | 1F | AACATCTGCTTTCCCGAGCC | 255-274 | 84 | 100 | |
| | | | | 1R | GCCGCCCTTCCATAGCATA | 338-320 | | | |
| | | | | 2F | GCAGTCGTCATGTCCCGAA | 58-76 | 81 | 105 | |
| | | | | 2R | ATGGATGCCCTCACTTGGAT | 138-119 | | | |
| Pro Kin | Protein kinase | AJ495779.1 | *Hordeum vulgare* | 1F | TGGAATCGTGATGTGGGAGC | 565-584 | 103 | 105 | |
| | | | | 1R | CAGAGGCCGTAAGGTGTTGT | 667-648 | | | |
| | | | | 2F | CTAAGAGATCCCCAACGCCC | 395-414 | 89 | 104 | |
| | | | | 2R | CGCACTCCACCAGAGATCAG | 483-464 | | | |
| | Protein kinase | MH551224.1 | | 1F | TTCAGCCATGTCAAAGGCCA | 50-69 | 108 | 103 | |

| | | | | | | | | |
|---|---|---|---|---|---|---|---|---|
| | | | *Triticum aestivum* | 1R | TAGTCATCCTGCTCACCCCA | 157-138 | | |
| | | | | 2F | GTCTTGGCTGCATGTTTGCT | 691-710 | 186 | 104 |
| | | | | 2R | TTCTGCTGTGCCTTCCAACA | 876-857 | | |
| Pro Phos | Protein Phosphatase 2B Regulatory Subunit (Calcineurin Regulatory Subunit) | BM816073.1 | *Hordeum vulgare* | 1F | GAAGAAGCCGGGTACACACA | 525-544 | 124 | 99 |
| | | | | 1R | ATCCACATTCGCCGTTCACT | 648-629 | | |
| | | | | 2F | CTGGTAGAGGTTTTGCGGGA | 447-466 | 99 | 99 |
| | | | | 2R | CTGTGTGTACCCGGCTTCTT | 545-526 | | |
| | Serine/threonine-protein phosphatase PP2A-1 catalytic subunit (PP2Ac-1) | EF101900 | *Triticum aestivum* | 1F | CGTGTCCAAGAGGTTCCACA | 611-630 | 98 | 105 |
| | | | | 1R | CCAGCACCACGAGGAGAAAT | 708-689 | | |
| | | | | 2F | CAGCCCTGGTGGAATCTGAA | 525-544 | 169 | 103 |
| | | | | 2R | GAAATGCCCCAACCACATCG | 693-674 | | |
| Myb | transcription factor GAMYB | AK251726.1 | *Hordeum vulgare* | 1F | GGGGACACTTCGTCTCATCC | 1729-1748 | 138 | 110 |
| | | | | 1R | GAAAACAGGCTTGCACTCCG | 1866-1847 | | |
| | | | | 2F | GAGTGCAAGCCTGTTTTCGG | 1849-1868 | 115 | 110 |
| | | | | 2R | TGGAGGTTCGGTGAGGAACT | 1963-1944 | | |
| | R2R3-MYB transcription factor (RIM1) | KU864997.1 | *Triticum aestivum* | 1F | ACAGCTTTTGGTCGGAGACA | 655-674 | 120 | 97 |
| | | | | 1R | AGTCCATCTCGTCGTTGCTG | 774-755 | | |
| | | | | 2F | GCCATGCTCGGCAATAGATG | 345-364 | 97 | 101 |
| | | | | 2R | GCTTCTTGAGGTGTGTGTGC | 441-422 | | |
| Gras | GRAS-domain transcription factor | AK371946.1 | *Hordeum vulgare* | 1F | CCTTCTTCATGCCCCTCTCG | 924-943 | 117 | 109 |
| | | | | 1R | GGTGGTTTCGGGTGAGGTAG | 1040-1021 | | |
| | | | | 2F | TCCCATGCTGTTCCACGAAG | 811-830 | 91 | 107 |
| | | | | 2R | GTGCTGGTGCTGATACTGGT | 901-882 | | |
| | GRAS transcription factor SHRCD | KT809308.1 | *Triticum aestivum* | 1F | GGCATGTTCCCCTCCGAC | 213-230 | 70 | 102 |
| | | | | 1R | GGTCCTTCCACCCGAGCTT | 282-264 | | |
| | | | | 2F | GGCGCAGTCCGGGTTC | 146-161 | 85 | 108 |

| | | | | | | | | | |
|---|---|---|---|---|---|---|---|---|---|
| | | | | 2R | GTCGGAGGGGAACATGCC | 230-213 | | | |
| Mads | MADS-box transcription factor (type II subfamily) | AK370732.1 | *Hordeum vulgare* | 1F | TATTTGAAGATGGGGCGGGG | 171-190 | 132 | 97 | |
| | | | | 1R | GGCATCGCAGAGAATACCCA | 302-283 | | | |
| | | | | 2F | CGGCAGTTGATGGGACAAGA | 501-520 | 163 | 107 | |
| | | | | 2R | CTTGGTGGACAAGACTCCCC | 663-644 | | | |
| | MADS box transcription factor | AB084577.1 | *Triticum aestivum* | 1F | CAACAGCGTGAAAGCAACCA | 304-323 | 97 | 108 | |
| | | | | 1R | CTGGTAGTGCTGGGCATTGA | 400-381 | | | |
| | | | | 2F | GGAGGGAAGGCTGGACAAAG | 508-527 | 156 | 102 | |
| | | | | 2R | TGTTGCCCCCTTTCAGTCTC | 663-644 | | | |
| **Reference Gene** | | | | Sequence (5'->3') | | | Amplicon size (bp) | Efficiency % | |
| TubB | beta-tubulin | | | F | CAAGGAGGTGGACGAGCAGATG | | 84 | 90 | [24] |
| | | | | R | GACTTGACGTTGTTGGGGATCCA | | | | |
| GAPDH | Glyceraldehyde-3-phosphate dehydrogenase | | | F | TGTCCATGCCATGACTGCAA | | 105 | 101 | |
| | | | | R | CCAGTGCTGCTTGGAATGATG | | | | |
| **Calibration gene** | | species | | Sequence (5'->3') | | position | Amplicon size (bp) | Efficiency % | |
| PVinterF | Coat protein | Luteovirus, Barley yellow dwarf virus | | F | GTTGAGTTTAAGTCACACGC | 3182-3201 | 294 | 99 | [24] |
| YanRA | | | | R | TGTTGAGGAGTCTACCTATTTG | 3475-3454 | | | [29] |

**Table S2**. Expression profile showing the fold change mean values, SEM and TTest.
(A. Barley 10 dai; B. Barley 30 dai; C. Wheat 10 dai; D. Wheat 30 dai)

| **A** | | **Graciosa 10 dai** | | **Travira 10 dai** | | **Wbon 10 dai** | | **Wysor 10 dai** | | **V08:3 _ 10 dai** | | **V13:8 _ 10 dai** | |
|---|---|---|---|---|---|---|---|---|---|---|---|---|---|
| Target gene | | Control | Infected | Control | Infected | Control | Infected | Control | Infected | Control | Infected | Control | Infected |
| *NBS* | AVERAGE | 1.000 | 3.843 | 1.000 | 1.230 | 1.000 | 0.767 | 1.000 | 0.874 | 1.000 | 0.855 | 1.000 | 1.787 |
| | SEM | 0.182 | 0.734 | 0.112 | 0.186 | 0.269 | 0.461 | 0.157 | 0.173 | 0.069 | 0.010 | 0.268 | 0.621 |
| | TTEST | | 0.020 | | 0.348 | | 0.665 | | 0.635 | | 0.203 | | 0.309 |
| *CC-NBS-LRR* | AVERAGE | 1.000 | 5.845 | 1.000 | 2.883 | 1.000 | 0.773 | 1.000 | 0.857 | 1.000 | 1.483 | 1.000 | 2.387 |
| | SEM | 0.116 | 0.691 | 0.068 | 0.717 | 0.298 | 0.200 | 0.131 | 0.190 | 0.097 | 0.169 | 0.224 | 0.804 |
| | TTEST | | 0.002 | | 0.059 | | 0.621 | | 0.561 | | 0.073 | | 0.172 |
| *Receptor-like Kinase* | AVERAGE | 1.000 | 7.021 | 1.000 | 3.266 | 1.000 | 0.815 | 1.000 | 0.472 | 1.000 | 1.089 | 1.000 | 2.166 |
| | SEM | 0.133 | 2.019 | 0.159 | 0.720 | 0.370 | 0.303 | 0.105 | 0.129 | 0.095 | 0.098 | 0.545 | 0.793 |
| | TTEST | | 0.041 | | 0.037 | | 0.749 | | 0.050 | | 0.578 | | 0.292 |
| *Casein Kinase* | AVERAGE | 1.000 | 1.723 | 1.000 | 2.181 | 1.000 | 0.442 | 1.000 | 1.158 | 1.000 | 1.641 | 1.000 | 0.969 |
| | SEM | 0.211 | 0.277 | 0.290 | 0.356 | 0.163 | 0.184 | 0.017 | 0.269 | 0.148 | 0.446 | 0.360 | 0.133 |
| | TTEST | | 0.106 | | 0.062 | | 0.113 | | 0.589 | | 0.193 | | 0.940 |
| *Protein Phosphatase* | AVERAGE | 1.000 | 1.268 | 1.000 | 1.664 | 1.000 | 0.510 | 1.000 | 0.877 | 1.000 | 0.949 | 1.000 | 0.754 |
| | SEM | 0.144 | 0.180 | 0.131 | 0.236 | 0.150 | 0.021 | 0.014 | 0.177 | 0.151 | 0.136 | 0.125 | 0.083 |
| | TTEST | | 0.309 | | 0.070 | | 0.085 | | 0.424 | | 0.831 | | 0.177 |
| *Protein Kinase* | AVERAGE | 1.000 | 2.769 | 1.000 | 1.481 | 1.000 | 0.475 | 1.000 | 1.027 | 1.000 | 1.232 | 1.000 | 1.099 |
| | SEM | 0.203 | 0.124 | 0.033 | 0.036 | 0.177 | 0.142 | 0.010 | 0.685 | 0.069 | 0.182 | 0.208 | 0.293 |
| | TTEST | | 0.002 | | 0.001 | | 0.129 | | 0.962 | | 0.250 | | 0.797 |
| *MYB TF* | AVERAGE | 1.000 | 4.540 | 1.000 | 0.852 | 1.000 | 0.704 | 1.000 | 0.549 | 1.000 | 0.723 | 1.000 | 0.850 |
| | SEM | 0.266 | 0.592 | 0.175 | 0.176 | 0.014 | 0.070 | 0.121 | 0.041 | 0.129 | 0.018 | 0.084 | 0.012 |
| | TTEST | | 0.004 | | 0.583 | | 0.013 | | 0.066 | | 0.195 | | 0.152 |
| *GRAS TF* | AVERAGE | 1.000 | 2.510 | 1.000 | 1.258 | 1.000 | 1.038 | 1.000 | 1.062 | 1.000 | 1.080 | 1.000 | 0.995 |
| | SEM | | 0.013 | 0.098 | 0.083 | 0.140 | 0.136 | 0.036 | 0.710 | 0.060 | 0.150 | 0.494 | 0.219 |
| | TTEST | | 0.001 | | 0.116 | | 0.866 | | 0.914 | | 0.598 | | 0.993 |
| *MADS-box TF* | AVERAGE | 1.000 | 4.540 | 1.000 | 2.458 | 1.000 | 0.739 | 1.000 | 1.764 | 1.000 | 1.444 | 1.000 | 1.832 |
| | SEM | 0.109 | 0.592 | 0.095 | 0.024 | 0.261 | 0.196 | 0.100 | 1.296 | 0.083 | 0.367 | 0.254 | 0.665 |
| | TTEST | | 0.888 | | 0.0001 | | 0.528 | | 0.490 | | 0.228 | | 0.307 |

| B Target gene | | Graciosa 30 dai | | Travira 30 dai | | Wbon 30 dai | | Wysor 30 dai | | V08:3 _ 30 dai | | V13:8 _ 30 dai | |
|---|---|---|---|---|---|---|---|---|---|---|---|---|---|
| | | Control | Infected | Control | Infected | Control | Infected | Control | Infected | Control | Infected | Control | Infected |
| *NBS* | AVERAGE | 1.000 | 3.314 | 1.000 | 2.082 | 1.000 | 1.623 | 1.000 | 1.208 | 1.000 | 0.898 | 1.000 | 0.563 |
| | SEM | 0.010 | 0.835 | 0.243 | 0.170 | 0.073 | 0.392 | 0.113 | 0.222 | 0.136 | 0.156 | 0.065 | 0.075 |
| | TTEST | | 0.050 | | 0.022 | | 0.194 | | 0.450 | | 0.648 | | 0.012 |
| *CC-NBS-LRR* | AVERAGE | 1.000 | 2.857 | 1.000 | 1.781 | 1.000 | 1.413 | 1.000 | 1.439 | 1.000 | 0.529 | 1.000 | 0.436 |
| | SEM | 0.165 | 0.324 | 0.152 | 0.414 | 0.181 | 0.230 | 0.111 | 0.103 | 0.295 | 0.133 | 0.035 | 0.084 |
| | TTEST | | 0.007 | | 0.152 | | 0.231 | | 0.044 | | 0.219 | | 0.003 |
| *Receptor-like Kinase* | AVERAGE | 1.000 | 10.429 | 1.000 | 2.677 | 1.000 | 0.814 | 1.000 | 0.402 | 1.000 | 0.204 | 1.000 | 0.347 |
| | SEM | 0.052 | 0.964 | 0.248 | 0.449 | 0.170 | 0.093 | 0.154 | 0.122 | 0.291 | 0.026 | 0.096 | 0.048 |
| | TTEST | | 0.001 | | 0.031 | | 0.392 | | 0.038 | | 0.053 | | 0.004 |
| *Casein Kinase* | AVERAGE | 1.000 | 2.621 | 1.000 | 1.908 | 1.000 | 1.953 | 1.000 | 1.129 | 1.000 | 0.248 | 1.000 | 1.012 |
| | SEM | 0.078 | 0.179 | 0.203 | 0.092 | 0.053 | 0.246 | 0.182 | 0.072 | 0.062 | 0.019 | 0.113 | 0.195 |
| | TTEST | | 0.001 | | 0.015 | | 0.019 | | 0.546 | | 0.064 | | 0.960 |
| *Protein Phosphatase* | AVERAGE | 1.000 | 1.504 | 1.000 | 1.109 | 1.000 | 1.291 | 1.000 | 1.348 | 1.000 | 1.418 | 1.000 | 0.702 |
| | SEM | 0.169 | 0.103 | 0.180 | 0.206 | 0.105 | 0.072 | 0.093 | 0.201 | 0.062 | 0.061 | 0.087 | 0.059 |
| | TTEST | | 0.063 | | 0.711 | | 0.083 | | 0.191 | | 0.009 | | 0.047 |
| *Protein Kinase* | AVERAGE | 1.000 | 1.775 | 1.000 | 1.252 | 1.000 | 1.538 | 1.000 | 1.524 | 1.000 | 1.488 | 1.000 | 0.878 |
| | SEM | 0.076 | 0.212 | 0.119 | 0.057 | 0.118 | 0.209 | 0.274 | 0.148 | 0.149 | 0.034 | 0.019 | 0.084 |
| | TTEST | | 0.026 | | 0.129 | | 0.088 | | 0.168 | | 0.033 | | 0.228 |
| *MYB TF* | AVERAGE | 1.000 | 2.106 | 1.000 | 1.498 | 1.000 | 1.105 | 1.000 | 1.038 | 1.000 | 0.598 | 1.000 | 0.849 |
| | SEM | 0.152 | 0.261 | 0.206 | 0.313 | 0.097 | 0.098 | 0.260 | 0.222 | 0.099 | 0.046 | 0.195 | 0.140 |
| | TTEST | | 0.022 | | 0.255 | | 0.489 | | 0.917 | | 0.021 | | 0.563 |
| *GRAS TF* | AVERAGE | 1.000 | 0.786 | 1.000 | 1.208 | 1.000 | 1.242 | 1.000 | 1.437 | 1.000 | 0.476 | 1.000 | 0.273 |
| | SEM | 0.229 | 0.039 | 0.232 | 0.166 | 0.071 | 0.208 | 0.485 | 0.371 | 0.298 | 0.056 | 0.159 | 0.056 |
| | TTEST | | 0.408 | | 0.507 | | 0.334 | | 0.514 | | 0.159 | | 0.013 |
| *MADS-box TF* | AVERAGE | 1.000 | 0.569 | 1.000 | 1.652 | 1.000 | 1.347 | 1.000 | 0.658 | 1.000 | 0.136 | 1.000 | 0.305 |
| | SEM | 0.243 | 0.047 | 0.339 | 0.233 | 0.250 | 0.537 | 0.632 | 0.169 | 0.408 | 0.019 | 0.167 | 0.155 |
| | TTEST | | 0.157 | | 0.188 | | 0.590 | | 0.629 | | 0.102 | | 0.169 |

| C | | Semper 10 dai | | SGS27-02 _ 10 dai | | Tobak 10 dai | | Sparta 10 dai | | Elan 10 dai | | PSR 3628 _ 10 dai | |
|---|---|---|---|---|---|---|---|---|---|---|---|---|---|
| Target gene | | Control | Infected | Control | Infected | Control | Infected | Control | Infected | Control | Infected | Control | Infected |
| *NBS* | AVERAGE | 1.000 | 31.864 | 1.000 | 18.342 | 1.000 | 7.319 | 1.000 | 5.890 | 1.000 | 2.098 | 1.000 | 1.887 |
| | SEM | 0.119 | 2.684 | 0.262 | 3.064 | 0.575 | 1.150 | 0.229 | 1.782 | 0.392 | 1.276 | 0.444 | 0.491 |
| | TTEST | | 0.0003 | | 0.030 | | 0.008 | | 0.053 | | 0.457 | | 0.251 |
| *CC-NBS-LRR* | AVERAGE | 1.000 | 13.875 | 1.000 | 2.478 | 1.000 | 10.377 | 1.000 | 4.617 | 1.000 | 2.542 | 1.000 | 0.765 |
| | SEM | 0.736 | 2.846 | 0.687 | 1.226 | 0.072 | 2.151 | 0.324 | 1.770 | 0.164 | 0.702 | 0.676 | 0.213 |
| | TTEST | | 0.012 | | 0.352 | | 0.010 | | 0.115 | | 0.124 | | 0.757 |
| *Receptor-like Kinase* | AVERAGE | 1.000 | 20.345 | 1.000 | 13.595 | 1.000 | 2.385 | 1.000 | 0.519 | 1.001 | 0.689 | 1.000 | 0.396 |
| | SEM | 0.354 | 2.165 | 0.353 | 4.307 | 0.210 | 0.518 | 0.137 | 0.091 | 0.222 | 0.153 | 0.204 | 0.042 |
| | TTEST | | 0.001 | | 0.043 | | 0.061 | | 0.043 | | 0.177 | | 0.044 |
| *Casein Kinase* | AVERAGE | 1.000 | 6.161 | 1.000 | 1.811 | 1.000 | 4.165 | 1.000 | 1.652 | 1.000 | 2.649 | 1.000 | 0.949 |
| | SEM | 0.037 | 1.907 | 0.071 | 0.290 | 0.003 | 0.398 | 0.106 | 0.108 | 0.575 | 0.101 | 0.483 | 0.196 |
| | TTEST | | 0.054 | | 0.053 | | 0.002 | | 0.012 | | 0.048 | | 0.927 |
| *Protein Phosphatase* | AVERAGE | 1.000 | 7.951 | 1.000 | 1.438 | 1.000 | 1.660 | 1.000 | 1.775 | 1.000 | 1.860 | 1.000 | 0.941 |
| | SEM | 0.256 | 0.992 | 0.141 | 0.166 | 0.076 | 0.019 | 0.264 | 0.305 | 0.144 | 0.445 | 0.470 | 0.001 |
| | TTEST | | 0.002 | | 0.115 | | 0.007 | | 0.127 | | 0.140 | | 0.906 |
| *Protein Kinase* | AVERAGE | 1.000 | 0.397 | 1.000 | 1.444 | 1.000 | 1.918 | 1.000 | 0.849 | 1.000 | 1.454 | 1.000 | 1.012 |
| | SEM | 0.070 | 0.063 | 0.143 | 0.062 | 0.045 | 0.321 | 0.099 | 0.130 | 0.335 | 0.439 | 0.405 | 0.056 |
| | TTEST | | 0.003 | | 0.047 | | 0.034 | | 0.407 | | 0.457 | | 0.978 |
| *MYB TF* | AVERAGE | 1.000 | 21.074 | 1.000 | 1.264 | 1.000 | 2.987 | 1.000 | 0.672 | 1.000 | 0.631 | 1.000 | 1.361 |
| | SEM | 0.054 | 2.078 | 0.060 | 0.326 | 0.230 | 0.083 | 0.093 | 0.148 | 0.173 | 0.081 | 0.175 | 0.282 |
| | TTEST | | 0.001 | | 0.470 | | 0.007 | | 0.135 | | 0.125 | | 0.338 |
| *GRAS TF* | AVERAGE | 1.000 | 19.726 | 1.000 | 1.208 | 1.000 | 5.585 | 1.000 | 0.914 | 1.000 | 0.535 | 1.000 | 1.258 |
| | SEM | 0.049 | 1.612 | 0.206 | 0.382 | 0.035 | 0.560 | 0.133 | 0.042 | 0.288 | 0.144 | 0.435 | 0.333 |
| | TTEST | | 0.0003 | | 0.656 | | 0.002 | | 0.568 | | 0.222 | | 0.663 |
| *MADS-box TF* | AVERAGE | 1.000 | 7.112 | 1.000 | 1.385 | 1.000 | 2.039 | 1.000 | 0.750 | 1.000 | 0.933 | 1.000 | 0.844 |
| | SEM | 0.303 | 4.337 | 0.035 | 0.195 | 0.043 | 0.627 | 0.232 | 0.243 | 0.438 | 0.317 | 0.378 | 0.046 |
| | TTEST | | 0.233 | | 0.124 | | 0.114 | | 0.499 | | 0.907 | | 0.702 |

| **D** Target gene | | **Semper 30 dai** | | **SGS27-02 _ 30 dai** | | **Tobak 30 dai** | | **Sparta 30 dai** | | **Elan 30 dai** | | **PSR 3628 _ 30 dai** | |
|---|---|---|---|---|---|---|---|---|---|---|---|---|---|
| | | Control | Infected | Control | Infected | Control | Infected | Control | Infected | Control | Infected | Control | Infected |
| *NBS* | AVERAGE | 1.000 | 30.701 | 1.000 | 1.822 | 1.000 | 1.143 | 1.000 | 0.524 | 1.000 | 4.629 | 1.000 | 0.310 |
| | SEM | 0.092 | 8.088 | 0.184 | 0.684 | 0.298 | 0.282 | 0.494 | 0.090 | 0.241 | 1.226 | 0.233 | 0.034 |
| | TTEST | | 0.016 | | 0.425 | | 0.707 | | 0.397 | | 0.044 | | 0.043 |
| *CC-NBS-LRR* | AVERAGE | 1.000 | 5.949 | 1.000 | 1.092 | 1.000 | 0.258 | 1.000 | 0.891 | 1.000 | 0.839 | 1.000 | 0.443 |
| | SEM | 0.019 | 0.899 | 0.095 | 0.163 | 0.044 | 0.022 | 0.072 | 0.038 | 0.104 | 0.133 | 0.136 | 0.030 |
| | TTEST | | 0.005 | | 0.861 | | 0.0001 | | 0.251 | | 0.193 | | 0.013 |
| *Receptor-like Kinase* | AVERAGE | 1.000 | 9.030 | 1.000 | 0.625 | 1.000 | 2.638 | 1.000 | 0.603 | 1.000 | 0.685 | 1.000 | 1.152 |
| | SEM | 0.034 | 0.868 | 0.135 | 0.105 | 0.159 | 0.936 | 0.024 | 0.106 | 0.137 | 0.081 | 0.130 | 0.282 |
| | TTEST | | 0.001 | | 0.094 | | 0.160 | | 0.011 | | 0.120 | | 0.650 |
| *Casein Kinase* | AVERAGE | 1.000 | 0.905 | 1.000 | 0.922 | 1.000 | 1.538 | 1.000 | 0.702 | 1.000 | 0.916 | 1.000 | 0.962 |
| | SEM | 0.018 | 0.158 | 0.179 | 0.132 | 0.034 | 0.227 | 0.149 | 0.088 | 0.110 | 0.051 | 0.125 | 0.262 |
| | TTEST | | 0.550 | | 0.743 | | 0.079 | | 0.160 | | 0.529 | | 0.901 |
| *Protein Phosphatase* | AVERAGE | 1.000 | 0.868 | 1.000 | 0.815 | 1.000 | 0.462 | 1.000 | 0.769 | 1.000 | 0.856 | 1.000 | 0.639 |
| | SEM | 0.002 | 0.467 | 0.208 | 0.102 | 0.003 | 0.070 | 0.304 | 0.089 | 0.028 | 0.009 | 0.102 | 0.088 |
| | TTEST | | 0.729 | | 0.468 | | 0.002 | | 0.506 | | 0.008 | | 0.055 |
| *Protein Kinase* | AVERAGE | 1.000 | 0.326 | 1.000 | 0.747 | 1.000 | 1.735 | 1.000 | 0.576 | 1.000 | 0.822 | 1.000 | 0.484 |
| | SEM | 0.008 | 0.045 | 0.147 | 0.113 | 0.071 | 0.222 | 0.159 | 0.026 | 0.101 | 0.068 | 0.155 | 0.027 |
| | TTEST | | 0.0001 | | 0.244 | | 0.034 | | 0.058 | | 0.214 | | 0.025 |
| *MYB TF* | AVERAGE | 1.000 | 2.617 | 1.000 | 0.534 | 1.000 | 0.475 | 1.000 | 0.706 | 1.000 | 0.595 | 1.000 | 2.195 |
| | SEM | 0.089 | 0.035 | 0.172 | 0.161 | 0.057 | 0.069 | 0.020 | 0.098 | 0.036 | 0.073 | 0.077 | 0.539 |
| | TTEST | | 0.001 | | 0.119 | | 0.004 | | 0.043 | | 0.008 | | 0.093 |
| *GRAS TF* | AVERAGE | 1.000 | 4.910 | 1.000 | 1.300 | 1.000 | 0.469 | 1.000 | 0.704 | 1.000 | 1.319 | 1.000 | 2.463 |
| | SEM | 0.012 | 1.614 | 0.330 | 0.071 | 0.155 | 0.117 | 0.135 | 0.245 | 0.085 | 0.073 | 0.037 | 0.530 |
| | TTEST | | 0.047 | | 0.423 | | 0.052 | | 0.349 | | 0.047 | | 0.051 |
| *MADS-box TF* | AVERAGE | 1.000 | 1.674 | 1.000 | 1.891 | 1.000 | 1.765 | 1.000 | 0.938 | 1.000 | 1.664 | 1.000 | 2.064 |
| | SEM | 0.060 | 0.617 | 0.270 | 0.352 | 0.086 | 0.224 | 0.080 | 0.294 | 0.171 | 0.121 | 0.029 | 0.735 |
| | TTEST | | 0.244 | | 0.115 | | 0.033 | | 0.716 | | 0.034 | | 0.221 |

**Figure S1.** BYDV-PAV titre for all barley and wheat samples with multifactorial ANOVA values.

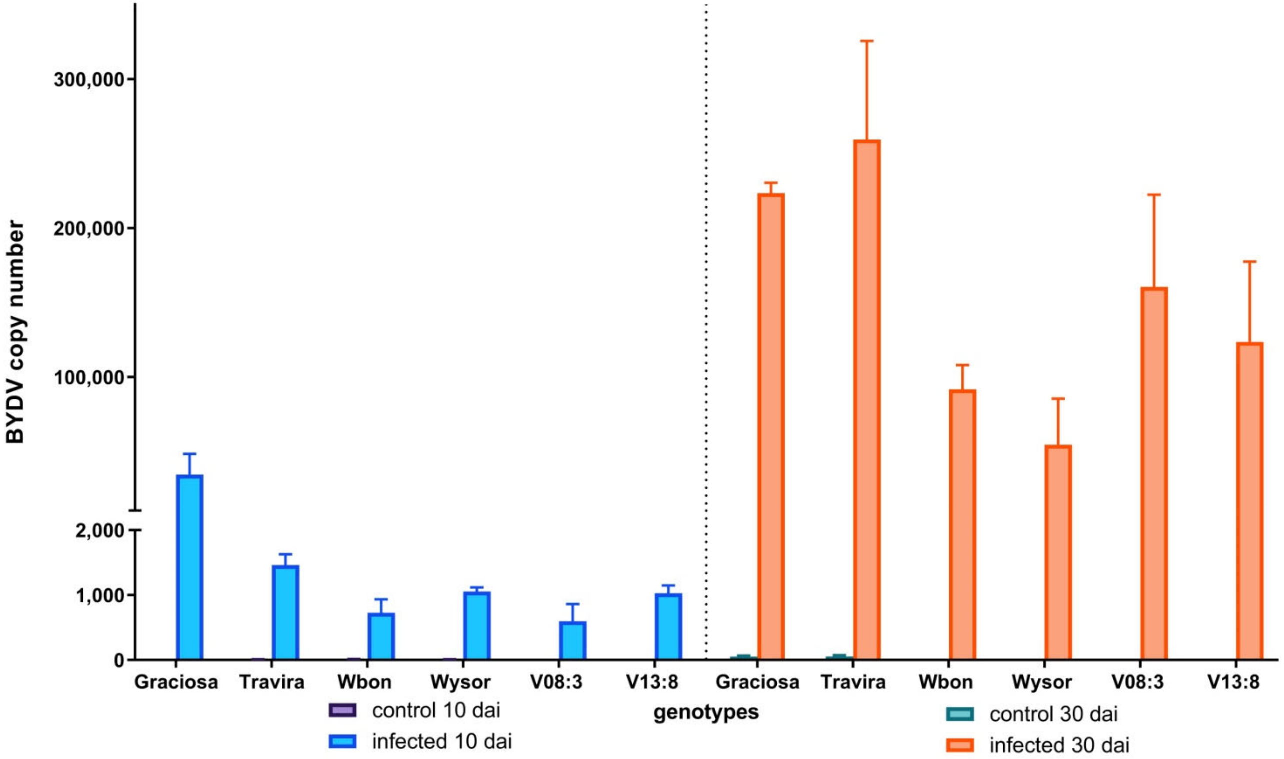


| ANOVA table | SS | DF | MS | F (DFn, DFd) | P value |
|---|---|---|---|---|---|
| genotype | 27361561784 | 5 | 5472312357 | F (5, 20) = 3.684 | P=0.0159 |
| control vs infected | 95414519993 | 1 | 95414519993 | F (1, 4) = 47.44 | P=0.0023 |
| 10 dai vs 30 dai | 113333938656 | 1 | 113333938656 | F (1, 4) = 56.34 | P=0.0017 |
| genotype x control vs infected | 20028080267 | 5 | 4005616053 | F (5, 20) = 2.696 | P=0.0509 |
| genotype x 10 dai vs 30 dai | 27335285120 | 5 | 5467057024 | F (5, 20) = 3.682 | P=0.0159 |
| control vs infected x 10 dai vs 30 dai | 95377246635 | 1 | 95377246635 | F (1, 4) = 47.42 | P=0.0023 |
| genotype x control vs infected x 10 dai vs 30 dai | 20006583648 | 5 | 4001316730 | F (5, 20) = 2.695 | P=0.0510 |
